# Metabolic reprogramming identifies the most aggressive lesions at early phases of hepatic carcinogenesis

**DOI:** 10.18632/oncotarget.8632

**Published:** 2016-04-07

**Authors:** Marta Anna Kowalik, Giulia Guzzo, Andrea Morandi, Andrea Perra, Silvia Menegon, Ionica Masgras, Elena Trevisan, Maria Maddalena Angioni, Francesca Fornari, Luca Quagliata, Giovanna Maria Ledda-Columbano, Laura Gramantieri, Luigi Terracciano, Silvia Giordano, Paola Chiarugi, Andrea Rasola, Amedeo Columbano

**Affiliations:** ^1^ Department of Biomedical Sciences, University of Cagliari, 09124, Cagliari, Italy; ^2^ Department of Biomedical Sciences, University of Padova, 35122, Padova, Italy; ^3^ Department of Experimental and Clinical Biomedical Sciences, University of Florence, 50134 Firenze and Tuscan Tumor Institute, Florence, Italy; ^4^ Department of Oncology, University of Torino School of Medicine, Candiolo Cancer Institute-FPO, IRCCS, 10060, Candiolo, Italy; ^5^ Azienda Ospedaliero-Universitaria Policlinico S. Orsola Malpighi, 40138, Bologna, Italy; ^6^ Molecular Pathology Division, Institute of Pathology, University Hospital of Basel, CH-4003, Basel, Switzerland

**Keywords:** TRAP1, NRF2, HCC, oxidative phosphorylation, pentose phosphate pathway

## Abstract

Metabolic changes are associated with cancer, but whether they are just bystander effects of deregulated oncogenic signaling pathways or characterize early phases of tumorigenesis remains unclear. Here we show in a rat model of hepatocarcinogenesis that early preneoplastic foci and nodules that progress towards hepatocellular carcinoma (HCC) are characterized both by inhibition of oxidative phosphorylation (OXPHOS) and by enhanced glucose utilization to fuel the pentose phosphate pathway (PPP). These changes respectively require increased expression of the mitochondrial chaperone TRAP1 and of the transcription factor NRF2 that induces the expression of the rate-limiting PPP enzyme glucose-6-phosphate dehydrogenase (G6PD), following miR-1 inhibition. Such metabolic rewiring exclusively identifies a subset of aggressive cytokeratin-19 positive preneoplastic hepatocytes and not slowly growing lesions. No such metabolic changes were observed during non-neoplastic liver regeneration occurring after two/third partial hepatectomy. TRAP1 silencing inhibited the colony forming ability of HCC cells while NRF2 silencing decreased G6PD expression and concomitantly increased miR-1; conversely, transfection with miR-1 mimic abolished G6PD expression. Finally, in human HCC patients increased G6PD expression levels correlates with grading, metastasis and poor prognosis. Our results demonstrate that the metabolic deregulation orchestrated by TRAP1 and NRF2 is an early event restricted to the more aggressive preneoplastic lesions.

## INTRODUCTION

Most neoplastic cells increase glucose utilization and uncouple it from oxygen availability to face hypoxic conditions that might occur in the core of a fast growing tumor mass [1]. This metabolic shift, termed aerobic glycolysis or Warburg effect [2, 3, 4], is also required to sustain the anabolic needs of tumor cells [5], mainly by conveying metabolites into the pentose phosphate pathway (PPP) that provides both biosynthetic building blocks and anti-oxidant defenses [6]. In parallel, mitochondria of tumor cells down-modulate oxidative phosphorylation (OXPHOS) [7] and enhance glutamine catabolism to fuel essential metabolic reactions, such as the tricarboxylic acid (TCA) cycle [8].

Although the precise mechanisms responsible for the complex rewiring of these circuitries remain poorly understood, several signaling molecules constitutively deregulated in tumors, such as the PI3K/Akt/mTOR pathway [9], p53 [10], HIF-1α [11] and Myc [12] directly promote OXPHOS down-modulation and increased utilization of both glutamine and glucose. Moreover, inhibition of miR-1 following induction of the transcription factor NRF2 and accumulation of glycolytic intermediates increase PPP activity [13, 14]. Tumor cells exploit several strategies to inhibit rate-limiting glycolytic enzymes: pyruvate formation is controlled by enhanced expression of the pyruvate kinase enzyme M2 (PKM2) [15]; phosphofructokinase 1 (PFK1) is inhibited either by induction of the fructose-2,6-bisphosphatase TP53-inducible glycolysis and apoptosis regulator TIGAR [16], or by increasing intracellular levels of citrate, a PFK1 allosteric inhibitor [17]. Citrate also contributes to down-regulation of mitochondrial OXPHOS by inhibiting succinate dehydrogenase (SDH), the complex II of the respiratory chain [17]. In some tumor types OXPHOS is also inhibited by increased expression of TRAP1, a mitochondrial molecular chaperone of the Hsp90 family [18] that down-regulates both respiratory complex IV [19] and SDH [20]. SDH inhibition prompts accumulation of succinate and the ensuing activation of the transcription factor HIF-1α [21]. In this scenario, succinate acts as an oncometabolite [22], implying that metabolic changes elicited by TRAP1 directly contribute to tumor growth [18].

In spite of these observations, key questions on the significance of metabolic alterations in cancer remain unsolved. Indeed, most of the experiments have been performed either on tumor cells *in vitro*, where their metabolic features can be altered, or on xenografts of cancer cell lines and fully developed cancers. Thus, it is still unknown whether a metabolic rewiring occurs in the early stages of neoplastic progression. This point is crucial to understand if metabolic alterations drive the tumorigenic process or are just bystander effects of deregulated oncogenic signaling pathways.

To address this question, in the present study we utilized the well-characterized rat Resistant-Hepatocyte (R-H) model of hepatocarcinogenesis, in which tumors are initiated by the chemical carcinogen diethylnitrosamine (DENA) and promoted by a brief treatment with 2-acetylaminofluorene (2-AAF) combined with partial hepatectomy (PH) [23]. The advantage of this model is that it offers the possibility to identify distinct lesions (preneoplastic foci, preneoplastic nodules, early and fully developed HCCs), at well-defined timings. Through this experimental model, we investigated (i) whether a shift from OXPHOS to glycolysis is an early event in the hepatocarcinogenic process; (ii) whether PPP activation plays a role in the onset and progression of the process and (iii) what are the key molecules involved in the metabolic reprogramming of preneoplastic lesions.

## RESULTS

### The switch from OXPHOS to glycolysis is a very early event in hepatocarcinogenesis

**Figure 1:**
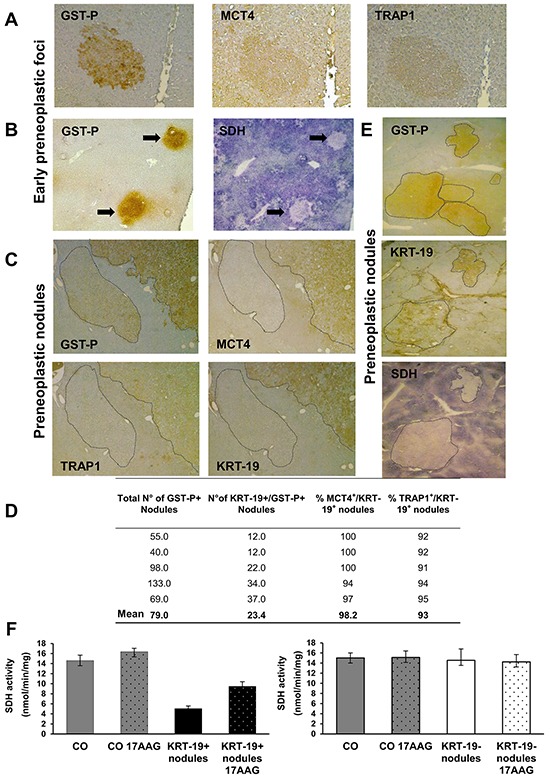
Induction of MCT4 and TRAP1 and inhibition of SDH in Early Preneoplastic Foci (EPF) and preneoplastic nodules **A.** IHC on serial sections of livers from rats sacrificed 4 weeks after DENA (1 week after PH) shows staining for both MCT4 and TRAP1 in a GST-P^+^ EPF (x10). **B.** Histochemical reaction showing impaired SDH activity in GST-P^+^ EPF (arrows) (x4). **C.** IHC on serial sections of livers from rats sacrificed 10 weeks after DENA shows MCT4 and TRAP1 staining in a GST-P^+^/KRT-19^+^ nodule. Absence of MCT4 and TRAP1 staining is observed in an adjacent GST-P^+^/KRT-19^−^ nodule (dotted area, x4). **D.** Percentage and number of KRT-19^+^/MCT4^+^ and KRT-19^+^/TRAP1^+^ nodules with respect to the total amount of KRT-19^+^ nodules. **E.** Photomicrographs showing a perfect match between GST-P/KRT-19 induction and SDH activity inhibition in nodules (dotted areas) (x1.25). **F.** SQR enzymatic activity of SDH in control livers (CO) and GST-P^+^/KRT-19^+^ (left) or GST-P^+^/KRT-19^−^ (right) nodules; when indicated, 17-AAG was added 5 min before starting recordings.

We investigated the metabolic features of early preneoplastic foci (EPF) in the RH model, characterized by a synchronous expansion of carcinogen-initiated cells that can be easily identified by preneoplastic markers, such as the glutathione S-transferase, placental form (GST-P), as early as 3-7 days after PH. Indeed, unlike normal hepatocytes, preneoplastic hepatocytes are able to rapidly divide after 2/3 PH, in the presence of the cytostatic environment generated by 2-AAF. EPF appear as small spherical lesions consisting of 15 to 100 basophilic hepatocytes, displaying several BrdU-positive nuclei and mitotic figures (Supplementary Figure S1A–S1B). Immunohistochemical analysis revealed an increase of both the lactate transporter MCT4 and of the mitochondrial chaperone TRAP1 (Figure 1A) in GST-P^+^ foci, compared to surrounding liver. Notably, in GST-P^+^ EPF, marked inhibition of SDH activity (Figure 1B) matched TRAP1 induction. Taken together these data raise the possibility that metabolic adaptations involving a decrease in OXPHOS activity and an enhanced glycolysis are early events in the hepatocarcinogenic process.

### The switch from OXPHOS to glycolysis identifies a subset of aggressive preneoplastic lesions

A rapid expansion of EPF to a nodular stage takes place within a few weeks [23]. While none of EPF is positive for the putative progenitor cell marker cytokeratin-19 (KRT-19), some of them become KRT-19^+^ during their progression to a nodular stage [24]. Notably, although KRT-19^+^ nodules represent a minority of the total preneoplastic nodules observed 10 weeks after treatment with DENA, 80-90% of HCCs arising in this model are KRT-19^+^ [25, 26]. This indicates that KRT-19^+^ lesions have an advantage in the progression to malignancy, while KRT-19^−^ nodules undergo spontaneous regression during the carcinogenic process [27].

To investigate whether the metabolic reprogramming observed in EPF is maintained along the carcinogenic process and to establish whether it involves all preneoplastic nodules or is unique to the most aggressive GST-P^+^/KRT-19^+^ lesions, we performed immunohistochemistry in both these preneoplastic populations. Similar to EPF, a strong induction of both MCT4 and TRAP1 was observed in preneoplastic nodules, demonstrating that these metabolic changes accompany the progression of the tumorigenic process. Interestingly, these changes occurred exclusively in GST-P^+^/KRT-19^+^ nodules (Figure 1C). QRT-PCR analysis on laser-microdissected nodules showed that the MCT4 increase was matched by its transcriptional induction, whereas that of TRAP1 occurred at a post-transcriptional level (Supplementary Figure S2A). Conversely, GST-P^+^/KRT-19^−^ nodules did not show any MCT4 or TRAP1 staining (Figure 1C). Of the 395 GST-P^+^ nodules examined, only 117 were KRT-19^+^, but, 98% and 96% of KRT-19^+^ nodules were also positive for MCT4 and TRAP1, respectively (Figure 1D), while no MCT4 or TRAP1 staining was found in KRT-19^−^ nodules (data not shown).

**Figure 2:**
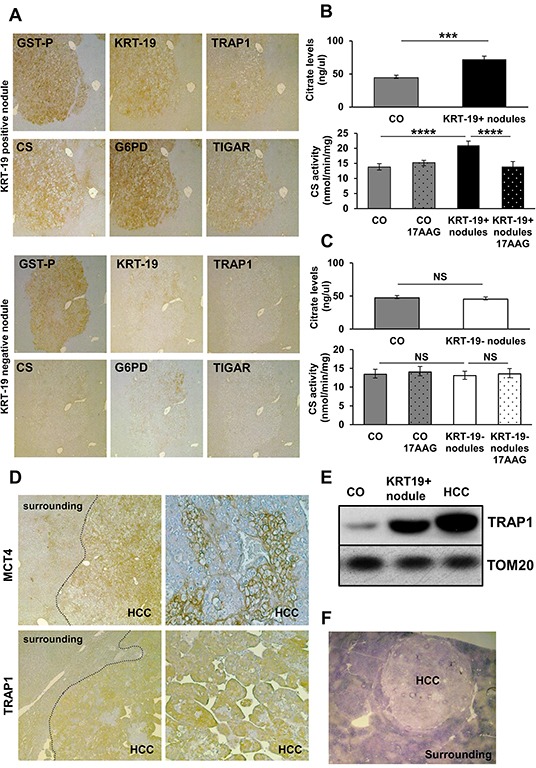
Analysis of metabolic markers in rat preneoplastic nodules and HCC **A.** IHC analysis on serial sections of liver nodules showing a perfect match between TRAP1, CS, G6PD and TIGAR and positivity for GST-P and KRT-19 (x4). **B–C.**  Analysis of citrate levels (top) and of CS activity (bottom) in control livers (CO) and KRT-19^+^ or KRT-19^−^ nodules treated or not with 17-AAG. ***P <0.001, ****P <0.0001. **D.** Staining of MCT4 and TRAP1 in HCCs, but not in the surrounding liver (x4); right side: higher magnification (x20) showing heterogeneous distribution of MCT4 and TRAP1 in HCC. **E.** Western immunoblot analysis of TRAP1 in KRT-19^+^ nodules, HCC and control liver (CO). TOM20: loading control. **F.** Photomicrograph showing impaired SDH activity in HCC (x1.25).

In line with what observed in EPF, TRAP1 induction matched inhibition of SDH activity only in preneoplastic GST-P^+^/KRT-19^+^ nodules (Figure 1E). Indeed, treatment with 17-AAG, a Hsp90/TRAP1 inhibitor that can be used as a *bona fide* selective TRAP1 inhibitor when measuring SDH activity [21], doubled SDH activity; SDH activity of GST-P^+^/KRT-19^−^ nodules was identical to that of normal liver, and 17-AAG was ineffective (Figure 1F). Moreover, a strong HIF-1α staining was observed only in preneoplastic GST-P^+^/KRT-19^+^ nodules (Supplementary Figure S2B). These data are in agreement with our previous observations showing that in tumor cells TRAP1 causes SDH inhibition, and the consequent rise in intracellular succinate levels induces HIF-1α stabilization [21]. GST-P^+^/KRT-19^+^ nodules also displayed a strong citrate synthase (CS) signal that paralleled TRAP1 induction (Figure 2A and Supplementary Figure S2C). Since citrate is an allosteric inhibitor of both PFK1 and SDH [17], enhancement of CS activity could play an important role in the metabolic rewiring of early lesions towards the inhibition of late glycolytic steps and OXPHOS. We therefore evaluated both citrate content and CS activity in macrodissected preneoplastic nodules and found that both were increased only in GST-P^+^/KRT-19^+^ lesions (Figure 2B–2C). Treatment with 17-AAG selectively inhibited CS activity in GST-P^+^/KRT-19^+^ nodules (Figure 2B–2C), suggesting that TRAP1 contributes to CS activation.

### Metabolic reprogramming is maintained in advanced HCCs

Fully advanced HCCs developed 14 months after treatment with DENA displayed several clusters of hepatocytes positive for both MCT4 and TRAP1 (Figure 2D), and an increase in TRAP1 protein levels in HCC, as well as in GST-P^+^/KRT-19^+^ nodules, was confirmed by Western blot analysis (Figure 2E). In accord with TRAP1 induction, a strong SDH activity inhibition was observed in HCC compared to the peri-tumoral tissue (Figure 2F). Thus, the metabolic reprogramming in very early stages of the neoplastic process is maintained in fully transformed cells, suggesting that it is a critical event in the progression of HCC development.

**Figure 3:**
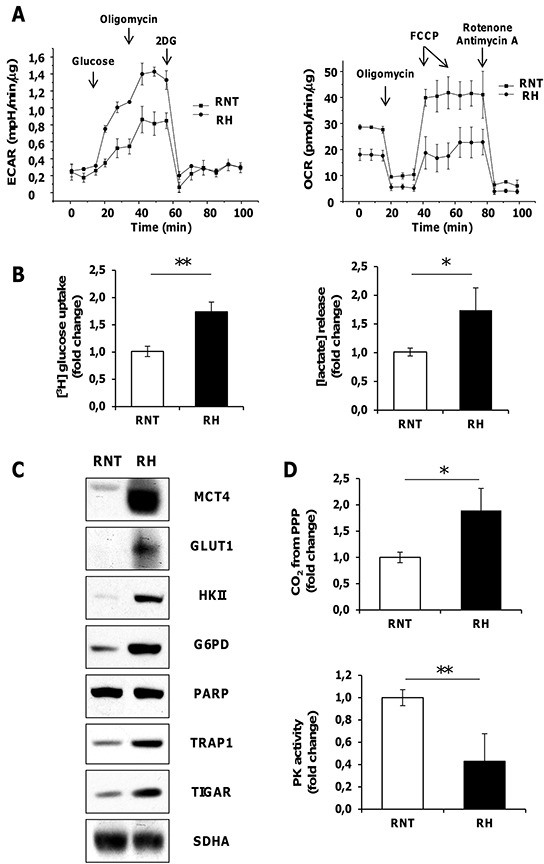
Rat HCC-derived cells (RH), unlike rat non tumorigenic hepatocytes (RNT) display high aerobic glycolytic activity, PPP activation and OXPHOS inhibition **A.** Measurements of extracellular acidification rate (ECAR, left) or of oxygen consumption rate (OCR, right) on monolayers of RNT and RH cells. Addition of D-Glucose, oligomycin, 2-Deoxy-D-glucose (right) and of oligomycin, FCCP, rotenone, and antimycin A (left) was carried out at the indicated times. **B.** Enhanced uptake of [^3^H] glucose and lactate release in RH cells. **C.** Western immunoblots showing MCT4, GLUT1, HK II, G6PD, TRAP1 and TIGAR expression. PARP and SDHA: loading controls. **D.** Radioactive assays using [^14^C]-glucose labeled in position C1 or in position C6 revealed an upregulation of CO_2_ produced from PPP; *P <0.05 (top); RH cells displayed a reduced PK enzymatic activity when compared to RNT cells (bottom). Data represent mean ± SEM **P <0.01D (left).

To further gain insights on the metabolic changes observed in hepatocarcinogenesis, HCC cells obtained from a HCC-bearing rat exposed to the RH protocol and sacrificed 14 months after DENA were compared to non-tumorigenic rat hepatocytes (RNT) derived from a rat exposed to the same protocol, with the exception of DENA (AAF + PH only); RNT cells did not acquire transformed features and maintained the normal hepatocyte morphology [26]. RH cells showed increased glycolytic activity, as they displayed an increase in the extracellular acidification rate (ECAR), which indicates enhanced lactate release following glucose administration (Figure 3A, left). The enhanced glycolytic activity of RH cells was also confirmed by their higher glucose uptake and lactate release and by an increase in the expression of both MCT4 and of the glucose transporter GLUT1, compared to RNT cells (Figure 3B-3C and Supplementary Figure S3A). In addition, the expression of Hexokinase II (HK II), the HK isoform highly induced in a variety of tumor cells [28], where it binds to mitochondria and contributes to cell survival [29, 30, 31], was much higher in RH cells than in RNT hepatocytes (Figure 3C), where HK II associated with mitochondria (Supplementary Figure S3B). In RH cells, glycolysis induction matched inhibition of OXPHOS, as demonstrated by decreased oxygen consumption rate (OCR) both in basal conditions and after maximal stimulation with the proton uncoupler carbonyl cyanide-4-(trifluoromethoxy) phenylhydrazone (FCCP) (Figure 3A, right). Moreover, RH cells had a diminished release of radiolabeled CO_2_ (Supplementary Figure S3C) and lower uptake of lactate (Supplementary Figure S3D). When we performed experiments using radioactive lactate, we found that signal of lactate-derived lipids and proteins paralleled that of lactate uptake and CO_2_ production from lactate (Supplementary Figure S3E). This suggests that the decrease observed in the lactate-dependent bioenergetic reactions and anabolism is due to the minor lactate uptake that characterizes the Warburg-addicted RH cells.

Compared to RNT cells RH cells expressed higher protein levels of TRAP1 (Figure 3C), and a lower succinate-coenzyme Q reductase (SQR) activity of SDH, that could be enhanced by 17-AAG, which was ineffective in RNT cells (Supplementary Figure S3F). in accord with a central role played by TRAP1 in energy metabolism of tumor cells, we found that knocking-down TRAP1 increased both basal and maximal oxygen consumption rate of RH cells (Supplementary Figure S4A). Moreover RH cells showed constitutive activation of HIF-1α (Supplementary Figure S4B), suggesting that TRAP1-dependent inhibition of SDH establish a pseudohypoxic phenotype and play a direct role in the tumorigenic process. Accordingly, we found that TRAP1 silencing inhibited the colony forming ability of RH cells, and this was re-established upon addition of a membrane-permeable succinate analogue (DMS) (Supplementary Figure S5A). Notably, TRAP1-silenced RH cancer cells displayed a reduced expression of both HK II and CS (Supplementary Figure S5B). These data, together with the observation that 17-AAG selectively impaired CS activity in GST-P^+^/KRT-19^+^ nodules (Figure 2B-2C), are in agreement with a role played by TRAP1 in favoring neoplastic progression by orchestrating both OXPHOS inhibition and glycolysis induction via SDH inhibition and CS induction.

### Diversion from glycolysis to PPP is restricted to KRT-19^+^ nodules

**Figure 4:**
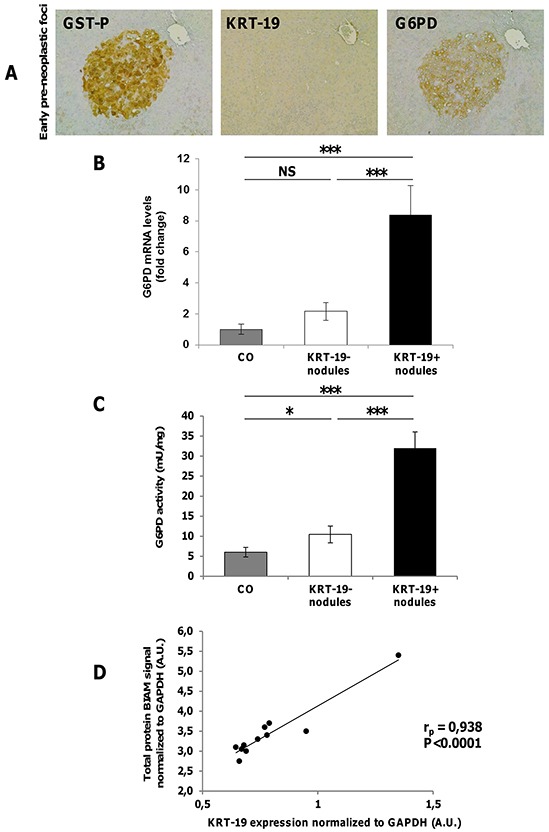
Diversion from glycolysis to PPP is restricted to highly proliferating GST-P^+^/KRT-19^+^ nodules **A.** IHC on serial sections of livers from rats sacrificed 4 weeks after DENA shows staining for G6PD in a GST-P^+^ EPF (x10). **B.** qRT-PCR analysis of G6PD mRNA in GST-P^+^/KRT-19^+^ and GST-P^+^/KRT-19^−^nodules. Gene expression is reported as fold-change relative to age-matched controls; ***P <0.001, NS: not significant. **C.** G6PD activity in GST-P^+^/KRT-19^+^ and GST-P^+^/KRT-19^−^nodules compared to controls; *P <0.05. **D.** Total lysates of 11 preneoplastic nodules were BIAM labeled and subjected to WB for KRT-19 or HRP-streptavidin. Streptavidin and KRT-19 signals were normalized to GAPDH expression and a correlation scatter plot generated. Statistical analysis revealed a positive correlation between Streptavidin signal (*i.e.* amount of reduced protein) and KRT-19 positivity.

Glucose can be diverted from glycolysis to PPP to supply anabolic components and anti-oxidant defenses to the rapidly growing tumor cells [6]. Therefore, we evaluated PPP induction in EPF and preneoplastic lesions. An increased expression of G6PD protein was observed in EPF, that are characterized by the absence of KRT-19 [24] (Figure 4A) and GSTP^+^-KRT-19^+^ (Figure 2A, top), but not in GSTP^+^-KRT-19^−^ nodules (Figure 2A, bottom). Enzymatic analysis showed that a strong increase of G6PD activity paralleled the enhanced levels of G6PD mRNA in GSTP^+^/KRT-19^+^ nodules (Figure 4B–4C).

PPP enhancement was maintained in RH cells, as shown by the increased amount of CO_2_ production from glucose radiolabeled in position 1 (CO_2_ derived from both OXPHOS and PPP) subtracting that originated from glucose radiolabeled in position 6 (CO_2_ exclusively derived from OXPHOS) (Figure 3D, top) and corroborated by G6PD enhanced expression (Figure 3C). Additionally, PPP induction was associated with inhibition of the activity of the final glycolytic enzyme pyruvate kinase (PK) (Figure 3D, bottom).

Additionally, fructose-2,6-bisphosphatase TP53-inducible glycolysis and apoptosis regulator (TIGAR) which decreases the level of fructose-2,6-bisphosphate, leading to inhibition of late glycolytic steps [16] and increasing glucose funneling into the PPP, was strongly induced in GSTP^+^/KRT19^+^ preneoplastic nodules, HCCs and RH cancer cells (Figure 2A-3C and Supplementary Figure S5C), but not in normal liver or GSTP^+^-KRT-19^−^ nodules.

PPP has a major role in ROS scavenging via NADPH synthesis [6]. Therefore, it has been proposed that enhanced PPP in neoplastic cells is required to support their anabolic demands and to combat oxidative stress [6]. To determine oxidative stress in early preneoplastic nodules, we labeled early nodules with BIAM to determine the overall amount of reduced proteins and found a strong positive Pearson correlation (r_p_=0.93, *P* < 0.0001) between KRT-19 and BIAM-labeled proteins (Figure 4D). This suggests that PPP induction observed in KRT19^+^ lesions may be a specific response to oxidants in aggressive preneoplastic cell populations.

### TRAP1 accumulation and G6PD up-regulation are not required for normal hepatocyte proliferation

**Figure 5:**
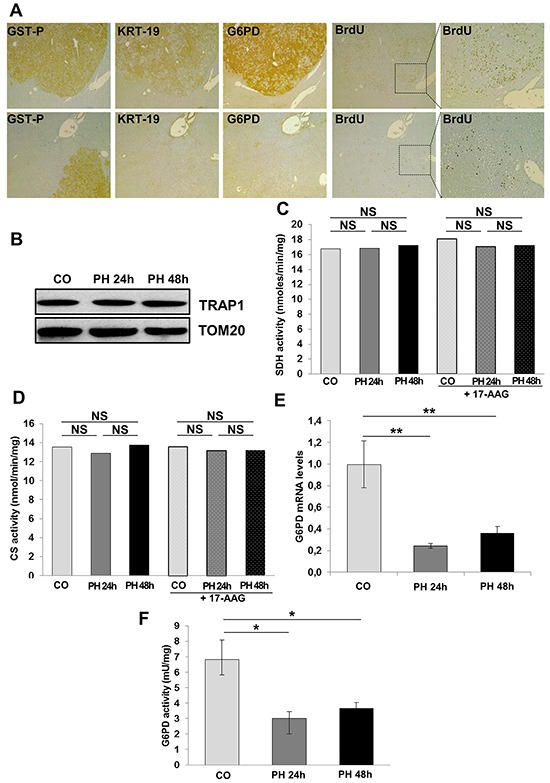
G6PD activity is not required for normal hepatocyte proliferation **A.** G6PD expression and BrdU incorporation in GST-P^+^/KRT-19^+^ nodules (x4). Inset; higher enlargement showing BrdU^+^ nuclei (x10). **B.** WB showing TRAP1 expression in quiescent and regenerating livers after 2/3 PH. TOM20: loading control. SDH (C) and CS (D) activities were assessed in control and regenerating liver post-PH; when indicated, samples were treated with 17-AAG. **E.** qRT-PCR analysis of G6PD mRNA in rats subjected to 2/3 PH and sacrificed 24 and 48 hours post-surgery. Gene expression is reported as fold-change relative to livers removed at the time of surgery; **P <0.01. **F.** G6PD activity in control and regenerating livers; *P <0.05.

Metabolic changes leading to increased G6PD expression are associated with a higher proliferative capacity of KRT-19^+^ preneoplastic lesions (Figure 5A). To determine whether induction of hepatocyte proliferation is sufficient *per se* to boost aerobic glycolysis and PPP or it is a feature unique to tumorigenesis, we studied the metabolic features of normal hepatocytes undergoing proliferation following 2/3 PH, an experimental model that causes massive hepatocyte replication. By comparing samples obtained 24 and 48 hours after PH (time of maximal DNA synthesis and of the second peak of hepatocyte proliferation, respectively) with quiescent liver specimens, we could not detect any change either in TRAP1 protein content (Figure 5B) or in SDH and CS enzymatic activities (Figure 5C–5D); moreover we observed a decrease of G6PD mRNA and activity (Figure 5E–5F). Analysis of BrdU-positive hepatocyte nuclei showed massive hepatocyte proliferation 24 hours after surgery, thus ruling out the possibility that the lack of increase of the examined parameters could be due to a lower number of proliferating cells in PH-livers compared to that of GST-P^+^/KRT19^+^ nodules (Supplementary Figure S6). These data demonstrate that a metabolic shift towards PPP induction and OXPHOS inhibition is not required for normal hepatocyte proliferation, but is characteristic of the tumorigenic process and takes place specifically in the most aggressive KRT-19^+^ preneoplastic lesions.

### NRF2 is required for the induction of G6PD, the TRAP1/HIF-1α pseudohypoxic axis and tumorigenicity

**Figure 6:**
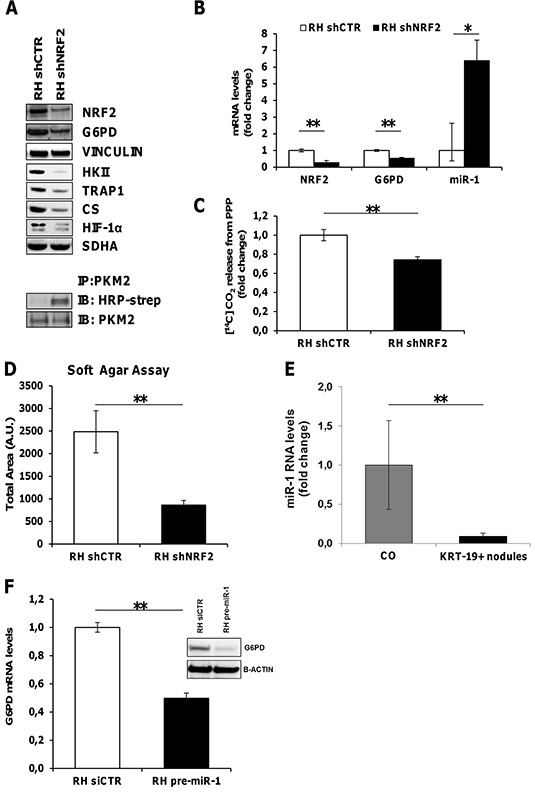
NRF2 modulates G6PD and miR-1 expression **A.** Top. WB showing inhibition of G6PD, HK II, TRAP1, CS and HIF-1α expression upon NRF2 silencing. Vinculin and SDHA: loading controls. Bottom. Immunoprecipitation assay showing an increase of the reduced form of PKM2 following NRF2 silencing in RH cells. **B.** qRT-PCR analysis of NRF2, G6PD and miR-1 RNA levels in RH cells upon NRF2 silencing. Values are reported as fold-change relative to cells transduced with a control shRNA. **C.** Radioactive assay using [^14^C]-glucose labeled in position C1 or in position C6 revealed a decrease in CO_2_ produced from PPP in RH cells silenced for NRF2 (shNRF2) **P <0.01. **D.** Soft agar tumorigenesis assays in RH cells following NRF2 silencing. Data indicate the total colony area at the 25th experimental day **P <0.01. **E.** qRT-PCR analysis of miR-1 expression in KRT-19^+^ nodules. Expression is reported as fold-change relative to age-matched controls; **P <0.01. **F.** qRT-PCR and WB analysis of G6PD expression in RH cells transfected with pre-miR-1.

PPP activation has recently been suggested as one mechanism by which deregulated NRF2/KEAP1 signaling promotes cellular proliferation and tumorigenesis [32, 33]. Since the NRF2/KEAP1 pathway is strongly activated in EPF and GST-P^+^/KRT-19^+^ nodules [25, 34], we investigated whether impairment of the NRF2 pathway could impact on G6PD expression levels. To this aim silencing of NRF2 was performed in RH tumorigenic cells. The results shown in Figure 6A-6B indicate that NRF2 silencing significantly decreased G6PD expression in RH tumorigenic cells and it was associated with a decrease of HK II, CS and HIF-1α protein levels (Figure 6A); while NRF2 silencing did not result in decreased levels of TRAP1 mRNA (Supplementary Figure S7), it caused a strong inhibition of TRAP1 protein content (Figure 6A); this result was somehow anticipated by the finding that TRAP1 protein content is not caused by increased transcription, but rather to post-translational mechanisms (Supplementary Figure S2A). Silencing of NRF2 also resulted in an increase of the reduced (less active) form of PKM2 (Figure 6A), and an impairment in the amount of glucose diverted into the PPP (Figure 6C). Therefore, NRF2 silencing causes inhibition of the TRAP1/HIF-1α pseudohypoxic axis and of glucose funneling into the PPP. These changes strongly impact on cell tumorigenicity, as shown by soft agar growth inhibition of NRF2-silenced RH cells (Figure 6D).

### miR-1 is involved in NRF2 induced activation of the PPP pathway

In agreement with recent findings proposing that NRF2 indirectly induces G6PD expression by down-regulating miR-1 [33], microarray analysis performed in microdissected preneoplastic KRT-19^+^ nodules showed an inverse correlation between miR-1 and its target gene, G6PD, (Supplementary Figure S8A). Comparable results were obtained by qRT-PCR validation analysis (Figure 6E). Accordingly, in NRF2-silenced RH cells, we observed an increased expression of miR-1, paralleled by G6PD down-regulation (Figure 6B). Conversely, RH cells transfected with pre-miR-1 showed a significant decrease of G6PD mRNA and protein levels (Figure 6F).

### G6PD expression is increased in human HCC

**Figure 7:**
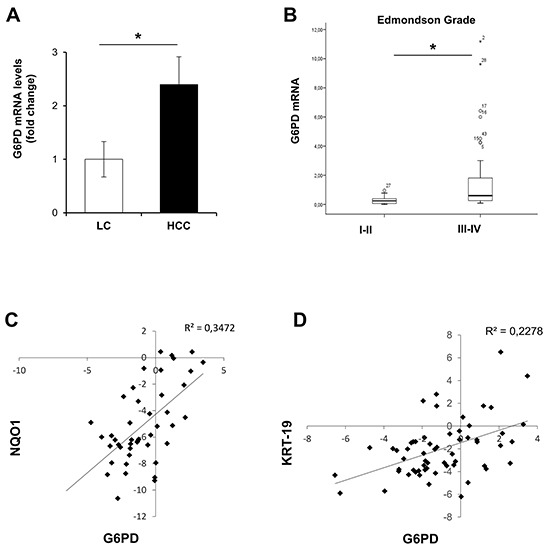
G6PD is up-regulated in human resected HCCs and correlates with NQO1 and KRT-19 mRNA levels **A.** Average levels of G6PD expression in human resected HCCs (n=59) and their peritumoural cirrhotic liver (Liver Cirrhosis, LC; n=59). G6PD mRNA expression is calculated as fold change difference compared to LC. β-actin was used as endogenous control; *P <0.05. **B.** G6PD mRNA levels correlate with high-grade (Edmonson III-IV), (*P =* 0.04). **C.** Scatter-plot graphic showing a positive correlation between NQO1 and G6PD mRNA levels in human resected HCCs; **D.** Scatter-plot graphic showing an positive correlation between KRT-19 and G6PD mRNA levels in the same human HCCs.

Having shown induction of G6PD and miR-1 down-regulation in both preneoplastic and neoplastic rat hepatocytes, we wished to determine whether these results could be of translational value for human HCC. Since it was not possible to collect a significant number of human early dysplastic lesions and in consideration of the finding that miR-1 and G6PD expression were dysregulated all throughout the rat carcinogenic process, we determined miR-1 expression and G6PD mRNA levels in a cohort of 59 patients subjected to liver resection for HCC (study population characteristics are described in Supplementary Table S1A). Similarly to what observed in rats, qRT-PCR analysis showed a significant down-regulation of miR-1 levels (Supplementary Figure S8B) (*P* <0.05) in 78% of HCCs, when compared to matched non-cancerous liver cirrhotic (LC) tissues. In agreement with the decreased miR-1 levels, we observed a concomitant increase of G6PD expression in the same human HCCs compared to LC *(P* <0.05) (Figure 7A). Higher G6PD expression was significantly associated to high-grade (Edmonson III-IV), aggressive tumors (*P* <0.05) (Figure 7B). No difference was observed between LC and non-cirrhotic liver (data not shown).

**Figure 8:**
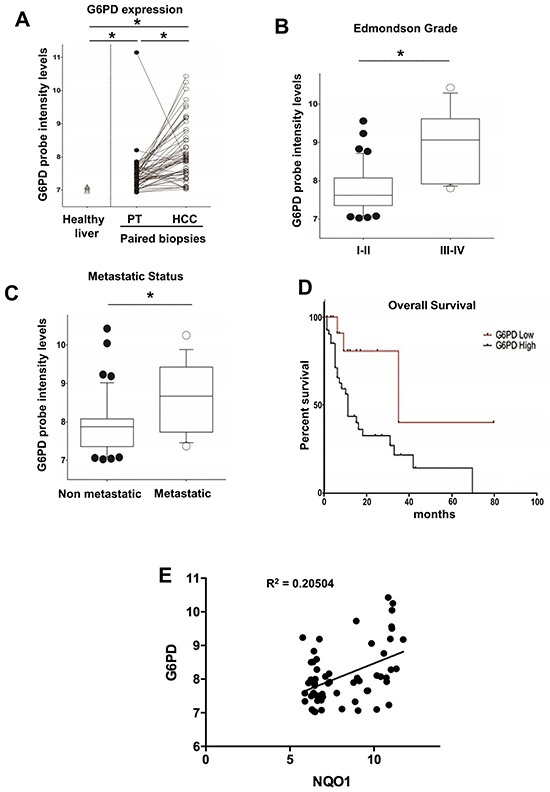
G6PD is up-regulated in biopsies from human HCCs and correlates with metastatic status, poor prognosis and NQO1 mRNA levels **A.** G6PD mRNA levels in a validating cohort of biopsied human HCCs (n=59) and their peritumoural liver (PT). The levels of G6PD mRNA in healthy livers are also shown (left); *P < 0.05. **B.** Higher G6PD expression was significantly associated with high-grade (Edmonson III-IV, *P <0.05) and significantly correlated **C.** with metastasis formation; *P <0.05; **D.** Kaplan-Maier analysis showing decreased overall survival of HCC patients expressing high G6PD mRNA levels; *P* = 0.0173. **E.** Scatter-plot graphic showing a positive correlation between NQO1 and G6PD mRNA levels in HCC.

To validate these data, we analyzed G6PD mRNA levels in a confirmatory independent cohort of 59 human HCCs and in the surrounding peritumoral tissues (study population characteristics are described in Supplementary Table S1B). Microarray analysis performed on liver biopsies obtained from patients carrying HCC confirmed a significant G6PD up-regulation in most of the tumors when compared to their peritumoral counterpart (*P* <0.05) (Figure 8A). Again, a higher G6PD expression was significantly associated to high-grade (Edmonson III-IV) aggressive tumors (*P* <0.05) (Figure 8B). Stratification of patients according to their etiology did not reveal any significant association with G6PD levels (Supplementary Figure S8C). Finally, to explore whether G6PD expression is associated with clinical progression and outcome, we examined the incidence of metastases and patient overall survival rates using Kaplan-Maier analysis. The metastatic status was defined as either regional lymph node invasion and/or distant organ involvement. High G6PD expression significantly correlated with metastasis formation (*P* <0.05) (Figure 8C) and decreased overall survival (median 35- and 11 months in low and high G6PD expressors), respectively (*P* =0.0173) (Figure 8D).

Notably, in both the cohorts a statistically significant increase of mRNA levels of NQO1, a well established NRF2-target gene, was found in HCC compared to cirrhotic peritumoral tissues. A significant positive correlation with the levels of G6PD mRNA was also observed in HCCs samples from patients subjected to liver resection (Figure 7C) or liver biopsies (Figure 8E). Furthermore, based on the strong link between KRT-19 expression and G6PD throughout HCC genesis in the rat model, we evaluated in the Bologna cohort of human HCC whether KRT-19 mRNA levels could be associated to G6PD expression. As shown in Figure 7D, a highly significant correlation between KRT-19 and G6PD (*P* < 0.001) was found between these two molecules.

## DISCUSSION

While a complex metabolic rewiring, mainly towards an enhanced glucose and glutamine utilization and a decrease in OXPHOS, has been shown in several types of cancer cells and is considered a hallmark of cancer [35, 36], it remains unknown whether it has a key role in the early stages of the tumorigenic process or is simply a bystander event of deregulated oncogenic signaling in fully transformed cells.

A novel finding of the present work is that this metabolic switch is a very early change, as it occurs concomitantly with the clonal expansion of carcinogen-induced “initiated” hepatocytes observed within few days. Notably, this metabolic reprogramming is selectively maintained only in KRT-19^+^ nodules that are considered the precursor lesions of HCC in this experimental model, and is exclusively associated to tumorigenesis and not merely linked to the entry into the cell cycle of normal hepatocytes. Interestingly, not all the cells within the nodules are homogeneously stained. This is particularly true for CS, TIGAR and TRAP1. Although, at the present it is not possible to fully appreciate the reason of this heterogeneity, this finding is expected as it clearly indicates that differences in the metabolic reprogramming occur inside the GST-P^+^/KRT-19^+^ nodules, and these probably depend on several environmental factors such as local oxygen tension or distance from capillaries.

Interestingly, this early metabolic shift is a two-pronged process composed of both OXPHOS inhibition and PPP induction. Two molecules seem to be deeply involved in this metabolic adjustment, TRAP1 and NRF2. TRAP1 is a mitochondrial molecular chaperone that is gaining increasing interest, as it orchestrates metabolic changes that influence neoplastic progression [18]. Indeed, TRAP1 knocking-down abolishes tumor growth both in *in vitro* assays and in xenografts [21]. Accordingly, in the present study TRAP1 induction was observed at all the stages of the tumorigenic process, namely in EPF, KRT-19^+^ nodules and full-blown HCC. The findings that i) TRAP1 silencing decreases HK II expression and *in vitro* tumorigenesis and ii) TRAP1 decreases SDH activity, prompting succinate accumulation and the ensuing HIF-1α stabilization, thus shifting the burden of ATP production to glycolysis [18, 21] are further indicative of a key role of this chaperone in the switch towards a more glycolytic, Warburg-like phenotype.

Additionally, TRAP1 can also have a role in reducing the glycolytic flux, hence inducing accumulation of glycolytic intermediates and their funneling into the PPP, by increasing CS protein level and enzymatic activity, leading to an increase of citrate, an important allosteric inhibitor of PFK1 and therefore of the late glycolytic steps [17].

The second branch of the metabolic rearrangement observed in the present study is the enhancement of PPP activity. Interestingly, both OXPHOS inhibition and the increased expression of G6PD, a rate-limiting enzyme in the PPP, were observed in EPF and in KRT-19^+^ nodules which are both characterized by a high proliferation rate. However, our present data demonstrate that G6PD, whose activity is required for providing both pentoses needed for the intense nucleic acid synthesis of dividing cells and NADPH to protect cells from increased ROS generation [6], is not a “*per se*” requirement for hepatocyte proliferation. Indeed, the massive hepatocyte proliferation observed during liver regeneration post-PH is associated with marked G6PD down-regulation. Thus, it is likely that the enhanced expression of this gene is specific for cells addressed to cancer progression and that the primary role of PPP induction is to maintain the redox equilibrium in preneoplastic cells, whereas pentoses can also be generated by the non-oxidative phase of PPP in a G6PD-independent manner [6]. Accordingly, we recently found that intracellular ROS levels are increased in tumorigenic cells compared to the non-tumorigenic counterpart [37]. In this context, it should be noted that the activation of NRF2/KEAP1 transcriptional pathway may play a critical role in G6PD increase. NRF2 regulates antioxidant genes, thus protecting cells from excessive ROS damage and, as recently shown, sustained activation of NRF2 signaling in cancer cells attenuated miR-1 expression, leading to enhanced expression of PPP genes [38]. Our study provides support to this finding and also demonstrates that, in an *in vivo* model of hepatocarcinogenesis, miR-1 expression is down-regulated in KRT-19^+^ nodules expressing high levels of NRF2-target genes [26]. Our data also show that NRF2 silencing decreased G6PD expression and concomitantly increased miR-1, while transfection with miR-1 mimic abolished G6PD expression. Moreover, NRF2 silencing down-modulated HK II, CS, TRAP1 and HIF-1α, further indicating a central function of NRF2 as metabolic programmer.

Remarkably, the translational value of the present results can be inferred by the observation that, similar to rat pre- and neoplastic lesions, in human HCCs high G6PD expression is associated with miR-1 down-regulation and correlates with tumor grading, metastatic status and poor prognosis. Finally, the relevance of these data is further strengthened by the concordance of the findings obtained in two large independent cohorts of human HCCs, made of either resected or liver biopsies samples.

In conclusion, the present work establishes that a metabolic shift characterized by impaired OXPHOS and increased glucose usage, mainly funneled into the PPP, is a very early event in hepatocarcinogenesis.

Future studies will address the question of whether targeting NRF2 impairs the growth of cancer cells and improve patients' prognosis and whether TRAP1 is another possible therapeutic target, as this chaperone might be responsible for the shift from OXPHOS to glycolysis.

## MATERIALS AND METHODS

### Animals and treatments

Guidelines for Care and Use of Laboratory Animals were followed during the investigation. All animal procedures were approved by the Ethical Commission of the University of Cagliari and the Italian Ministry of Health. Male Fischer rats (Charles River, Milano, Italy) were treated with a single dose of DENA (150 mg/kg) and, two weeks later, subjected to the RH protocol, consisting of a 2 weeks-diet supplemented with 0.02% 2-AAF and a 2/3 PH [23]. A group of animals was sacrificed 7 days after PH, while the remaining rats were then switched to basal diet and sacrificed ten weeks or fourteen months after DENA administration.

### Cell cultures and *in vitro* experiments

RH and RNT cells were obtained from rats treated with DENA+AAF+PH (tumor bearing rats) or AAF+PH (rats with no tumors), respectively, as described [26]. Briefly, liver was perfused at 10 ml/min via portal vein at 37°C for 5 minutes with buffer containing 1.9 mg/ml EGTA, for 2 minutes with buffer lacking EGTA, and for 10-15 minutes with buffer containing 0.03% (w/v) collagenase and 5 mM CaCl_2_. 2H_2_O as originally described [39] with modification for rat liver. The perfusion buffer contained 10 mM HEPES, 3 mM KCl, 130 mM NaCl, 1 mM NaH_2_PO_4_.H_2_O, and 10 mM D-glucose, pH 7.4 (Sigma-Aldrich; collagenase from Worthington Biochemical Corp.). The livers were dissociated in perfusion buffer, and cells were passed through Dacron fabric with 80 μm pores and centrifuged under 50 x *g* for 5 minutes to recover hepatocytes present in the pellet. For livers containing tumors, digestion continued in the plastic dish for an additional 10-20 min in the presence of collagenase: after incubation, the tumors present in the remaining parenchyma were mechanically disrupted. Cells were recovered and washed in medium several time, then seeded on collagen-coated plastic dish and maintained in RPMI 1640 medium supplemented with 10% FBS.

RH cells were transiently transfected with 200 pmol of miR-1 mimic (Ambion, Austin, TX) or 200 pmol of siRNAs (control siRNA or NRF2 siRNA, Ambion) using Lipofectamine 2000 (Invitrogen, Paisley, UK). RH cells were stably transduced with the 217CS-SH213J-LVRH1P lentiviral vector containing a specific custom-designed NRF2 targeting short hairpin RNA (GeneCopoeia, Rockville, MD). Transduced cells were selected by treatment with puromycin (1 μg/ml for 7 days; Invitrogen).

### Histology, immunohistochemistry and laser capture microdissection

Paraffin-embedded tissue was cut into 4 μm sections, dewaxed, and hydrated. Endogenous peroxide was inactivated using hydrogen peroxide. Slides were microwaved in citrate buffer pH 6.0 (Abcam), except for GST-P and TRAP1 staining, followed by overnight incubation with the primary antibodies: GSTP (MBL), KRT-19, HIF-1α (Novus Biologicals), MCT4, TRAP1, G6PD (Santa Cruz Biotechnology), CS (Abcam) and TIGAR (Millipore). After washes, the sections were incubated with the appropriate biotin-conjugated secondary antibody (Vector Laboratories) at room temperature. Signal was detected using the Vectastain ABC Elite kit (Vector Laboratories) and developed using 3,3′-diaminobenzidine tetrahydrochloride hydrate, DAB, (Sigma-Aldrich). Sections were counterstained with Harris hematoxylin solution (Sigma-Aldrich) and passed through the dehydration process and covered. For negative control, the sections were incubated with secondary antibodies only. For BrdU staining four-micrometer-thick sections were deparaffinized, treated with 2N HCl, then immersed in 0.1% Trypsin (Sigma-Aldrich) and incubated sequentially with goat serum (Dako), mouse monoclonal anti-BrdU antibody (Becton Dickinson) and with Dako EnVision+® System Labelled Polymer-HRP anti-mouse (Dako). The sites of peroxidase binding were detected by DAB.

KRT-19 and GSTP staining was also performed on frozen tissue as follows: cryostat cut (Leica CM 1950), frozen liver sections were fixed in −20°C acetone or in formalin at room temperature. Anti-CK19 antibody (Novocastra) or anti-GST-P antibody (MBL) were successively applied. After washes, the sections were incubated with the appropriate secondary antibodies (Sigma-Aldrich) at room temperature. The final reaction was visualized using DAB.

GSTP-positive, KRT-19-positive nodules were identified by immunohistochemical staining of 6 μm-thick frozen liver sections. Nodule microdissection was performed on 16 μm serial sections with a Leica LMD6000, as previously described [24].

### Immunoprecipitation, immunoblot analysis, antibodies and BIAM labeling

Cells derived from our experimental conditions were lysed in radioimmunoprecipitation assay (RIPA) buffer and 30 μg of total proteins were loaded on precast SDS-PAGE gels (BioRad), separated and transferred onto PVDF membrane. Immunoblots were incubated overnight in 5% BSA or milk in T-PBS at 4°C and probed with primary and appropriate secondary Horseradish Peroxidase (HRP)-antibodies. Immunoprecipitation was performed on 500 mg of total proteins in 500 ml of RIPA buffer using 20 μl of protein A/G plus (Santa Cruz Biotechnology) per samples, following manufacturer's instruction.

Detection of PKM2 redox state, N-(biotinoyl)-N’-(iodoacetyl) ethylene diamine BIAM labeling was performed as previously described [34]. Briefly, cells were rapidly rinsed in liquid nitrogen and exposed to RIPA buffer containing 100 μM of BIAM diamine and incubated for 15 min at room temperature. Lysates were then clarified by centrifugation and 500 μg of each sample were suspended into 500 μl of RIPA buffer and immunoprecipitated using 20 μl of Streptavidin-agarose beads (Sigma). Immunocomplexes labelled with BIAM were separated by SDS–PAGE and the biotinylated/reduced fraction was revealed with HRP-conjugated.

### Antibodies

Mouse monoclonal anti TIGAR and SDHA, goat polyclonal anti calnexin, actin and HK II, and rabbit polyclonal anti PARP, MCT4, G6PD and TOM20 antibodies were from Santa Cruz Biotechnology; rabbit polyclonal anti AIF and HIF1α antibodies were from Exalpha Biologicals and Novus Biologicals, respectively; rabbit polyclonal anti Citrate synthase and GLUT1 antibodies were from Abcam; mouse monoclonal anti GAPDH and TRAP1 antibodies were from Millipore and Becton Dickinson, respectively.

### RNA extraction and qRT-PCR

Total RNA was extracted from preneoplastic lesions, HCCs and RH cells with the MirVana kit (Life Technologies) and stored at −80°C until needed. Following extraction, RNA was quantified by NanoDrop ND1000 (Thermo Scientific), while RNA integrity was assessed by Agilent Bioanalyzer 2100. Only RNA samples with a RIN (RNA Integrity Number) ≥ 7 were included in the study. For HCCs and human HCC cell lines, RNA was extracted with RNeasy Plus Mini Kit (QIAGEN) or with TRIzol RNA isolation reagent (Life Technologies). RNA was retrotranscribed with High Capacity cDNA Reverse Transcription Kit (Applied Biosystems, Life Technologies) using random primers. Gene expression was evaluated by real time PCR analysis with an ABI PRISM 7300HT thermocycler (Applied Biosystems) on 16 samples of GSTP-positive preneoplastic nodules.

### Assay of intracellular reactive oxygen species (ROS)

To evaluate intracellular production of H_2_O_2_ cells were treated for 3 minutes with 5 μg/ml H2DCF-DA. After PBS washing, cells were lysed in RIPA buffer and analyzed immediately by fluorimetric analysis at 510 nm. Data were normalized to total protein content.

### Mitochondria fractionation

Mitochondria were isolated after cell disruption with a glass-Teflon or electrical potter (Sigma) in a buffer composed of 250 mM sucrose, 10 mM Tris-HCl, 0.1 mM EGTA-Tris, pH 7.4. Nuclei and plasma membrane fractions were separated by mild centrifugation (700 x *g*, 10 min); mitochondria were then spinned down at 7,000 x *g*, 10 min, and washed twice (7,000 x *g*, 10 min each). All procedures were carried out at 4°C.

### Measurements of oxygen consumption rate (OCR) and extracellular acidification rate (ECAR)

OCR was assessed in real-time with the XF24 Extracellular Flux Analyzer (Seahorse Biosciences), which allows to measure OCR changes after up to four sequential additions of compounds. Cells (6×10^4^/well) were plated the day before the experiment in a DMEM/10% serum medium; experiments were carried out on confluent monolayers. Before starting measurements, cells were placed in a running DMEM medium (supplemented with 25 mM glucose, 2 mM glutamine, 1 mM sodium pyruvate, and without serum and sodium bicarbonate) and pre-incubated for 1 hour at 37°C in atmospheric CO_2_. An accurate titration with the uncoupler FCCP was performed for each cell type, in order to utilize the FCCP concentration that maximally increases OCR. Together with OCR measurements, values of ECAR were also recorded as the absence of sodium bicarbonate allows changes in the pH of the medium. Addition of D-Glucose (10 mM), the ATP synthase inhibitor oligomycin (0.8 μM), the glycolytic inhibitor 2-Deoxy-D-glucose (2DG, 100 mM), the proton uncoupler FCCP (1 μM followed by 500 nM to a final concentration of 1.5 μM), the respiratory complex I inhibitor rotenone (1 μM), and the respiratory complex III inhibitor antimycin A (1 μM) was carried out at the times indicated.

### Pyruvate kinase (PK) activity

PK activity was measured by a double reaction kinetic assay. The PK-dependent conversion of PEP into pyruvate is followed by pyruvate conversion into lactate using lactate dehydrogenase (LDH), with a concomitant oxidation of NADH to NAD^+^ and a decrease in absorbance at 340 nm. Both reactions can be run simultaneously as a kinetic assay. Cells were lysed in 50 mM Tris-HCl pH 7.4, in the presence of protease inhibitors and degassed for 15 min. Measurement of PK activity was performed in 500 ml TRAP/4 (100 mM triethanolamine, 10 μM EDTA, 16 mM MgSO_4_, pH 7.6) in the presence of 15 μl ADP 100 mM, 15 μl NADH 10 mM, 20 μl PEP 40 mM, 1 μl LDH (Sigma L2500) and 10 μl of cell lysate (1 μg/ml), adjusting with H_2_O to a final volume of 1 μl. The absorbance at 340 nm was monitored over 20 min (ε= 6.22 mM^−1^xcm^−1^).

### Succinate dehydrogenase (SDH) activity

To measure the succinate-coenzyme Q reductase (SQR) enzymatic activity of respiratory chain complex II/succinate dehydrogenase (SDH), cells or liver samples were homogenized with a potter in a buffer composed by 250 mM sucrose, 10 mM Tris-HCl, 0.1 mM EGTA-Tris, pH 7.4, Percoll 10%, protease and phosphatase inhibitors and, when specified, mitochondria were isolated as described above. Cell homogenates or mitochondrial enriched fractions (20-40 mg per trace) were used for assaying the succinate-coenzyme Q reductase (SQR) activity of complex II/SDH through spectrophotometric recordings following the reduction of 2,6-dichlorophenolindophenol (DCPIP) at 600 nM (ε=19.1xmM^−1^xcm^−1^). Samples were pre-incubated for 10 min at 30°C in a buffer composed of 25 mM potassium phosphate pH 7.2, 20 mM sodium succinate, and 5 μM alamethicin. After the pre-incubation time, sodium azide (5 μM), antimycin A (2 μM), rotenone (2 μM) and DCPIP (50 μM) were added for 1 min to the medium. Reaction was performed at 30°C and started after the addition of an intermediate electron acceptor (Coenzyme Q1, 65 μM). Values were then normalized for protein amount.

Complex II/SDH enzymatic activity was also assessed histochemically on frozen tissue sections. Eight-micrometer-thick sections were incubated at 37°C in incubation medium, containing 0.2 M phosphate buffer, sodium succinate solution, NBT solution (Sigma-Aldrich) and dH_2_O. Successively, the liver tissue was rinsed in physiological saline, fixed in 10% formalin-saline solution, rinsed in 15% alcohol and finally mounted with an aqueous mounting medium. As succinate was oxidized to fumarate the reduced form of NADH was produced, and the reaction was visualized by chemically reacting the electron acceptor with the purple salt nitro blue tetrazolium (NBT).

### Glucose 6 phosphate dehydrogenase (G6PD) activity

Liver samples were pottered and lysed at 4°C in a buffer composed of 140 mM NaCl, 20 mM Tris-HCl pH 7.4, 5 mM EDTA, 10% glycerol, 1% Triton X-100, in the presence of phosphatase and protease inhibitors (Sigma). Lysates (10 μg) were then incubated with glucose-6-phosphate (1 mM) and NADP^+^ (1.5 mM). The rate of NADPH formation, which is proportional to the G6PD activity, was measured spectrophotometrically as an increase in absorbance at 340 nm at 30°C. Maleimide (12 mM) was added as an inhibitor of 6-phosphogluconate dehydrogenase.

### Citrate synthase (CS) activity

To measure CS activity, citrate formation was spectrophotometrically determined as an increase in absorbance at 420 nm at 37°C in a reaction buffer composed of 100 mM Tris-HCl pH 8, 0.1% Triton X-100, 100 μM 5,5b 2-dithiobis-(2-nitrobenzoic acid) (DTNB), 300 μM Acetyl -CoA, and 500 μM Oxaloacetate. Samples were prepared as described in the assay for SDH activity.

### Measurement of citrate levels

Citrate concentration was quantified with a coupled enzyme assay, which results in a colorimetric (570 nm)/fluorometric (λ_ex_ = 535/λ_em_ = 587 nm) product, proportional to the citrate present, following manufacturer's instructions (Sigma Aldrich kit MAK057). Briefly, samples were rapidly homogenized with 100 μl of the Citrate Assay Buffer (MAK057A) and then centrifuged (15000 x *g*, 10 minutes) to remove insoluble material; proteins were then removed with a 10 kDa MWCO spin filter and then samples were resuspended in Citrate Assay Buffer.

### Radioactive assays

Radioactive assays were performed as previously described [34]. Briefly, glucose or lactate uptake was evaluated in a buffered solution (140 mM NaCl, 20 mM Hepes/Na, 2.5 mM MgSO_4_, 1 mMCaCl_2_, and 5 mM KCl, pH 7.4) containing 0.5 μCi/ml [3H] deoxy-glucose or [U-^14^C] lactate for 15 minutes at 37°C. Cells were subsequently washed with cold PBS and lysed with 0.1 M NaOH. Detection of released CO_2_ by radioactive lactate and/or glucose was performed as a read out of OXPHOS activity when using [U-^14^C] glucose. PPP activity was evaluated by using radioactive glucose labeled in position 6 [6-^14^C] or in position 1 [1-^14^C]. ^14^CO_2_ developed from [1-^14^C]-glucose oxidation originates by the PPP or by the TCA cycle, whereas ^14^CO_2_ released from [6-^14^C]-glucose originates only by TCA cycle. 2 μCi [1-^14^C]-glucose or 2 μCi [6-^14^C]-glucose were added for 1 hour to cells (two different plates of same sample treated in parallel). PPP CO_2_ production is therefore revealed by subtracting the radioactive signal derived from [1-^14^C]-glucose to that of [6-^14^C]-glucose. Such values were then normalized on total cell protein content and shown as fold change. Each dish had a taped piece of Whatman paper facing the inside part of the dish wetted with 200 μL of phenyl-ethylamine-methanol (1:1) to trap the CO_2_. Then 150 μL of 4M H_2_SO_4_ was added to cells. Finally, Whatman paper was removed and transferred to scintillation vials for counting. Radioactive signal was measured by liquid scintillation counting and normalized for protein content.

### Incorporation of lactate into proteins or lipids

[U-^14^C] lactate was added to cell medium for 24 hours. For protein extraction, cells were resuspended in 20% trichloroacetic acid, placed on ice for 30 minutes and centrifuged. The resuspended pellet was assayed for [^14^C] labelled proteins by scintillator. For lipid extraction, cells were lysed in RIPA buffer and subjected to chloroform/methanol Bligh and Dyer extraction. The organic phase was recovered and assayed for [^14^C] labelled proteins by scintillator

### Soft agar assay

For soft agar assays cells were grown in 24 wells Petri dishes covered by a bottom layer composed of DMEM medium mixed with low melting point agarose (Promega) at a final concentration of 1.0%, and by a top layer of DMEM medium supplemented with 2% serum and mixed with low melting point agarose at a final concentration of 0.6%. Cells (1.5×10^4^) were added during the preparation of the upper layer, where they remained embedded. Dishes were then maintained in a humidified atmosphere of 5% CO_2_-95% air at 37°C for four weeks, changing the medium (DMEM 2% serum) on the top of the two layers every 3^th^ day. At the 30^th^ day, dishes were washed in PBS and colonies were stained with Crystal Violet 0.005% and analyzed with ImageJ software.

### Patients

Two cohorts of patients carrying HCC were examined. The first cohort consisted of HCC and cirrhotic tissues obtained from 59 consecutive patients (45 males and 14 females, median age ± SD: 65.2 ± 7.9, range 49-80 years) undergoing liver resection for HCC at the Department of Surgery of the University of Bologna. Eight normal liver tissues were obtained from patients undergoing liver surgery for traumatic lesions (5 cases) or haemangioma resection (3 cases). No patient received anticancer treatment prior to surgery. The characteristics of patients are described in Supplementary Table S1. All patients gave written informed consent to the study, which was approved by the Ethics Committee of the University of Bologna. In the second cohort, patient's specimens and clinico-pathological data were obtained from the Institute of Pathology, University Hospital of Basel, Switzerland. All patients gave written informed consent to the study, which was approved by the Ethics Committee of the University Hospital of Basel (EKKB). HCC diagnosis was verified by pathological examination, no anti-cancer treatments were given before biopsy collection. Tumor differentiation was defined according to Edmondson's grading system. Only biopsies containing at least 50% of tumor cells and no necrotic area were used in this study. The clinico-pathologic features of these samples are described in Supplementary Table S1.

### Human microarray analysis

RNA for the microarray for Transcriptomic profiling was isolated from 59 HCC needle biopsies matched with their corresponding non-neoplastic liver parenchyma and 5 normal liver donors. RNA was isolated with a Direct-Zol RNA MiniPrep Kit (Zymo Research) including on-column DNAse treatment. RNA concentration was assessed using Quibit (Invitrogen) and RNA integrity was monitored on the Bioanalyzer 2100 using the RNA6000 Chip (Agilent). 270 ng of DNAse-treated total RNA was subjected to target synthesis using the WT Expression kit (Ambion) following standard recommendations. Fragmentation and labelling of amplified cDNA were performed using the WT Terminal Labelling Kit (Affymetrix). Synthesis reactions were carried out using a PCR machine (TProfessionnalTrio, Biometra) in 0.2 ml tubes. 85 μl cocktail (23.4 ng/ ml labelled DNA) were loaded on GeneChip®Human Gene 1.0ST arrays (Affymetrix) and hybridized for 17 hours (45°C, 60 rpm) in the Hybridization oven 645 (Affymetrix). The arrays were washed and stained on the Fluidics Stations 450 (Affymetrix) by using the Hybridization Wash and Stain Kit (Affymetrix) under FS450_0002 protocol. The GeneChips were scanned with an Affymetrix GeneChip Scanner 3000 7G. DAT images and CEL files of the microarrays were generated using the Affymetrix GeneChip Command Control (version 4.0). Afterwards, CEL files were imported into Qlucore software and Robust Multichip Average (RMA) normalized. Subsequently, principal component analysis (PCA) to discriminate between normal and tumour samples was applied. Quantile normalization and data processing were performed using the GeneSpringGXv11.5.1 software package (Agilent). The gene signature value was assessed using the BRB-ArrayTool (v4.3.2, NIH). Ingenuity software (Qiagen, Hilden, Germany) was use to perform pathways analysis.

### Statistical analysis

Data are expressed as mean ± standard deviation (SD) or mean± standard error (SEM). Analysis of significance was performed by Student's *t* test and by One-Way ANOVA with Bonferroni correction when necessary.

## SUPPLEMENTARY FIGURES AND TABLE





## References

[1] Ward PS, Thompson CB (2012). Metabolic reprogramming: a cancer hallmark even warburg did not anticipate. Cancer Cell.

[2] Hsu PP, Sabatini DM (2008). Cancer cell metabolism: Warburg and beyond. Cell.

[3] Vander Heiden MG, Cantley LC, Thompson CB (2009). Understanding the Warburg effect: the metabolic requirements of cell proliferation. Science.

[4] Warburg O (1956). On the origin of cancer cells. Science.

[5] Schulze A, Harris AL (2012). How cancer metabolism is tuned for proliferation and vulnerable to disruption. Nature.

[6] Patra KC, Hay N (2014). The pentose phosphate pathway and cancer. Trends Biochem Sci.

[7] Gaude E, Frezza C (2014). Defects in mitochondrial metabolism and cancer. Cancer & Metabolism.

[8] Hensley CT, Wasti AT, DeBerardinis RJ (2013). Glutamine and cancer: cell biology, physiology, and clinical opportunities. J Clin Investi.

[9] Levine AJ, Puzio-Kuter AM (2010). The control of the metabolic switch in cancers by oncogenes and tumor suppressor genes. Science.

[10] Vousden KH, Ryan KM (2009). p53 and metabolism. Nat Rev Cancer.

[11] Semenza GL (2013). HIF-1 mediates metabolic responses to intratumoral hypoxia and oncogenic mutations. J Clin Invest.

[12] Dang CV (2012). MYC on the path to cancer. Cell.

[13] Hayes JD, Dinkova-Kostova AT (2014). The Nrf2 regulatory network provides an interface between redox and intermediary metabolism. Trends Biochem Sci.

[14] Jaramillo MC, Zhang DD (2013). The emerging role of the Nrf2-Keap1 signaling pathway in cancer. Genes & Development.

[15] Li Z, Yang P, Li Z (2014). The multifaceted regulation and functions of PKM2 in tumor progression. Biochim Biophys Acta.

[16] Lee P, Vousden KH, Cheung EC (2014). TIGAR, TIGAR, burning bright. Cancer & Metabolism.

[17] Icard P, Poulain L, Lincet H (2012). Understanding the central role of citrate in the metabolism of cancer cells. Biochim Biophys Acta.

[18] Rasola A, Neckers L, Picard D (2014). Mitochondrial oxidative phosphorylation TRAP(1)ped in tumor cells. Trends Cell Biol.

[19] Yoshida S, Tsutsumi S, Muhlebach G, Sourbier C, Lee MJ, Lee S, Vartholomaiou E, Tatokoro M, Beebe K, Miyajima N, Mohney RP, Chen Y, Hasumi H (2013). Molecular chaperone TRAP1 regulates a metabolic switch between mitochondrial respiration and aerobic glycolysis. Proc Natl Acad Sci USA.

[20] Guzzo G, Sciacovelli M, Bernardi P, Rasola A (2014). Inhibition of succinate dehydrogenase by the mitochondrial chaperone TRAP1 has anti-oxidant and anti-apoptotic effects on tumor cells. Oncotarget.

[21] Sciacovelli M, Guzzo G, Morello V, Frezza C, Zheng L, Nannini N, Calabrese F, Laudiero G, Esposito F, Landriscina M, Defilippi P, Bernardi P, Rasola A (2013). The mitochondrial chaperone TRAP1 promotes neoplastic growth by inhibiting succinate dehydrogenase. Cell Metab.

[22] Yang M, Soga T, Pollard PJ (2013). Oncometabolites: linking altered metabolism with cancer. J Clin Invest.

[23] Solt DB, Medline A, Farber E (1977). Rapid emergence of carcinogen-induced hyperplastic lesions in a new model for the sequential analysis of liver carcinogenesis. The American J Pathol.

[24] Kowalik MA, Sulas P, Ledda-Columbano GM, Giordano S, Columbano A, Perra A (2015). Cytokeratin-19 positivity is acquired along cancer progression and does not predict cell origin in rat hepatocarcinogenesis. Oncotarget.

[25] Andersen JB, Loi R, Perra A, Factor VM, Ledda-Columbano GM, Columbano A, Thorgeirsson SS (2010). Progenitor-derived hepatocellular carcinoma model in the rat. Hepatology.

[26] Petrelli A, Perra A, Cora D, Sulas P, Menegon S, Manca C, Migliore C, Kowalik MA, Ledda-Columbano GM, Giordano S, Columbano A (2014). MicroRNA/gene profiling unveils early molecular changes and nuclear factor erythroid related factor 2 (NRF2) activation in a rat model recapitulating human hepatocellular carcinoma (HCC). Hepatology.

[27] Enomoto K, Farber E (1982). Kinetics of phenotypic maturation of remodeling of hyperplastic nodules during liver carcinogenesis. Cancer Res.

[28] Mathupala SP, Ko YH, Pedersen PL (2010). The pivotal roles of mitochondria in cancer: Warburg and beyond and encouraging prospects for effective therapies. Biochim Biophys Acta.

[29] Chiara F, Castellaro D, Marin O, Petronilli V, Brusilow WS, Juhaszova M, Sollott SJ, Forte M, Bernardi P, Rasola A (2008). Hexokinase II detachment from mitochondria triggers apoptosis through the permeability transition pore independent of voltage-dependent anion channels. PloS One.

[30] Masgras I, Rasola A, Bernardi P (2012). Induction of the permeability transition pore in cells depleted of mitochondrial DNA. Biochim Biophys Acta.

[31] Pantic B, Trevisan E, Citta A, Rigobello MP, Marin O, Bernardi P, Salvatori S, Rasola A (2013). Myotonic dystrophy protein kinase (DMPK) prevents ROS-induced cell death by assembling a hexokinase II-Src complex on the mitochondrial surface. Cell Death Dis.

[32] Mitsuishi Y, Taguchi K, Kawatani Y, Shibata T, Nukiwa T, Aburatani H, Yamamoto M, Motohashi H (2012). Nrf2 redirects glucose and glutamine into anabolic pathways in metabolic reprogramming. Cancer Cell.

[33] Singh A, Happel C, Manna SK, Acquaah-Mensah G, Carrerero J, Kumar S, Nasipuri P, Krausz KW, Wakabayashi N, Dewi R, Boros LG, Gonzalez FJ, Gabrielson E (2013). Transcription factor NRF2 regulates miR-1 and miR-206 to drive tumorigenesis. J Clin Invest.

[34] Giannoni E, Taddei ML, Morandi A, Comito G, Calvani M, Bianchini F, Richichi B, Raugei G, Wong N, Tang D, Chiarugi P (2015). Targeting stromal-induced pyruvate kinase M2 nuclear translocation impairs oxphos and prostate cancer metastatic spread. Oncotarget.

[35] Hanahan D, Weinberg RA (2011). Hallmarks of cancer: the next generation. Cell.

[36] Pavlova NN, Thompson CB (2016). The Emerging Hallmarks of Cancer Metabolism. Cell Metab.

[37] Mattu S, Fornari F, Quagliata L, Perra A, Angioni MM, Petrelli A, Menegon S, Morandi A, Chiarugi P, Ledda-Columbano GM, Gramantieri L, Terracciano L, Giordano S (2016). The metabolic gene HAO2 is down regulated in mouse, rat and human hepatocellular carcinoma and correlates with metastasis and poor survival. J Hepatol.

[38] Zavattari P, Perra A, Menegon S, Kowalik MA, Petrelli A, Angioni MM, Follenzi A, Quagliata L, Ledda-Columbano GM, Terracciano L, Giordano S, Columbano A (2015). Nrf2, but not beta-catenin, mutation represents an early event in rat hepatocarcinogenesis. Hepatology.

[39] Follenzi A, Benten D, Novikoff P, Faulkner L, Raut S, Gupta S (2008). Transplanted endothelial cells repopulate the liver endothelium and correct the phenotype of hemophilia A mice. J Clin Invest.

