# Supplementary material for: Metabolic reprogramming identifies the most aggressive lesions at early phases of hepatic carcinogenesis
Source: Oncotarget. 2016 Apr 7;7(22):32375–93. doi: 10.18632/oncotarget.8632 (PMC5078020; doi:10.18632/oncotarget.8632)
Supplement: Supplementary file 1 [file oncotarget-07-32375-s001.pdf]

# Metabolic reprogramming identifies the most aggressive lesions at early phases of hepatic carcinogenesis

## SUPPLEMENTARY FIGURES AND TABLE

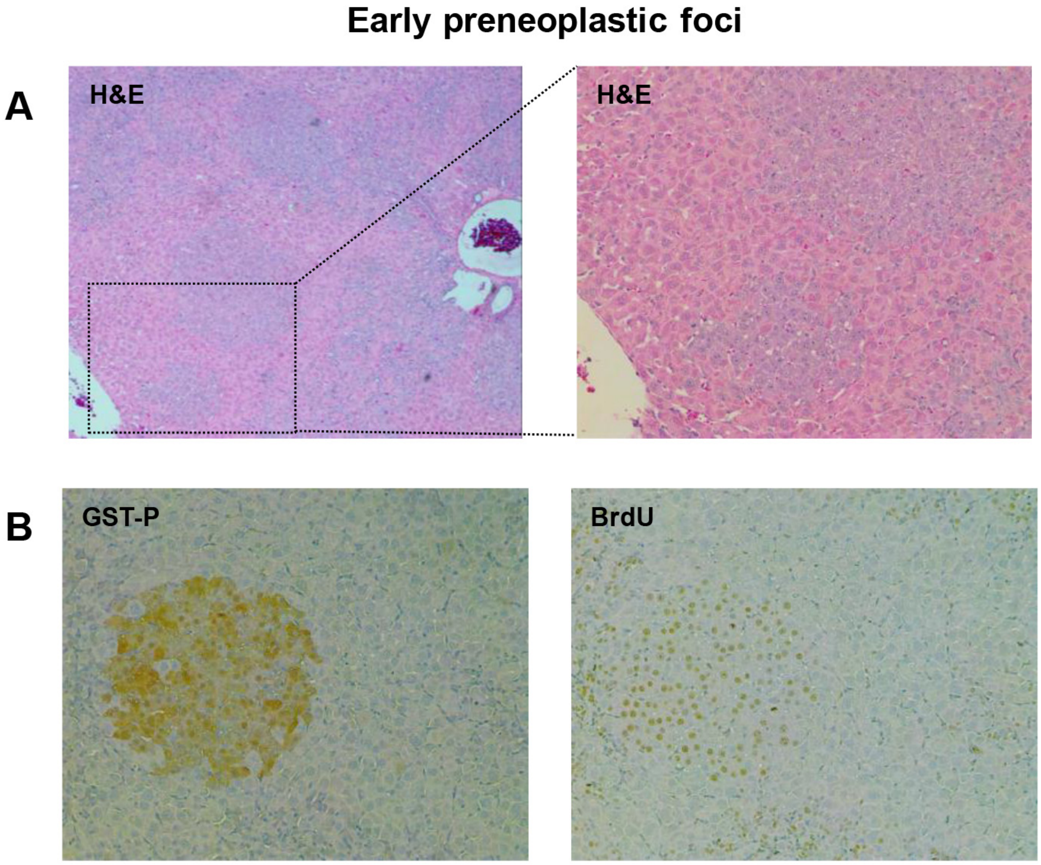


**Supplementary Figure S1: Strong proliferative activity in early preneoplastic foci. A.** H&E staining of basophilic foci developed 1 week after PH (x4). Inset: higher enlargement (x10). **B.** Microphotograph showing that most of nuclei are stained for BrdU (right) in a GSTP+ EPFs (x10). Several mitoses are present. BrdU was given in drinking water for 24 hours prior to sacrifice.

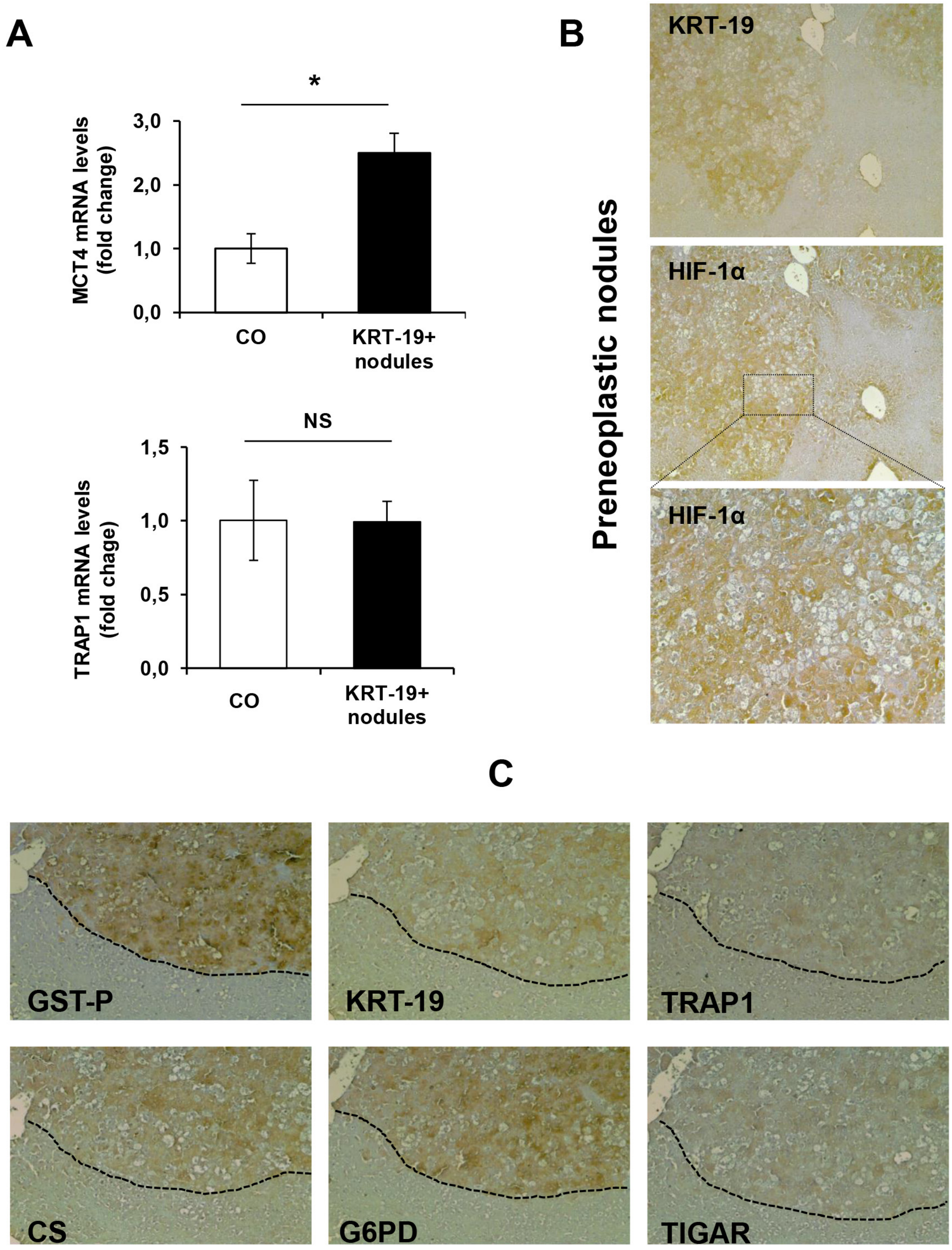


**Supplementary Figure S2: Analysis of markers of metabolic change in preneoplastic nodules. A.** qRT-PCR analysis of MCT4 and TRAP1 in laser-microdissected GST-P$^+$ preneoplastic nodules. Gene expression is reported as fold-change relative to age-matched controls; *P<0.05, NS: not significant. **B.** Strong HIF-1α expression in a KRT-19$^+$ nodule (x4). Inset: enlargement showing nuclear HIF-1α staining (x10). **C.** IHC analysis on serial sections of liver nodules showing an heterogeneous staining for TRAP1, CS and TIGAR in GST-P$^+$/KRT-19+ nodules (x20).

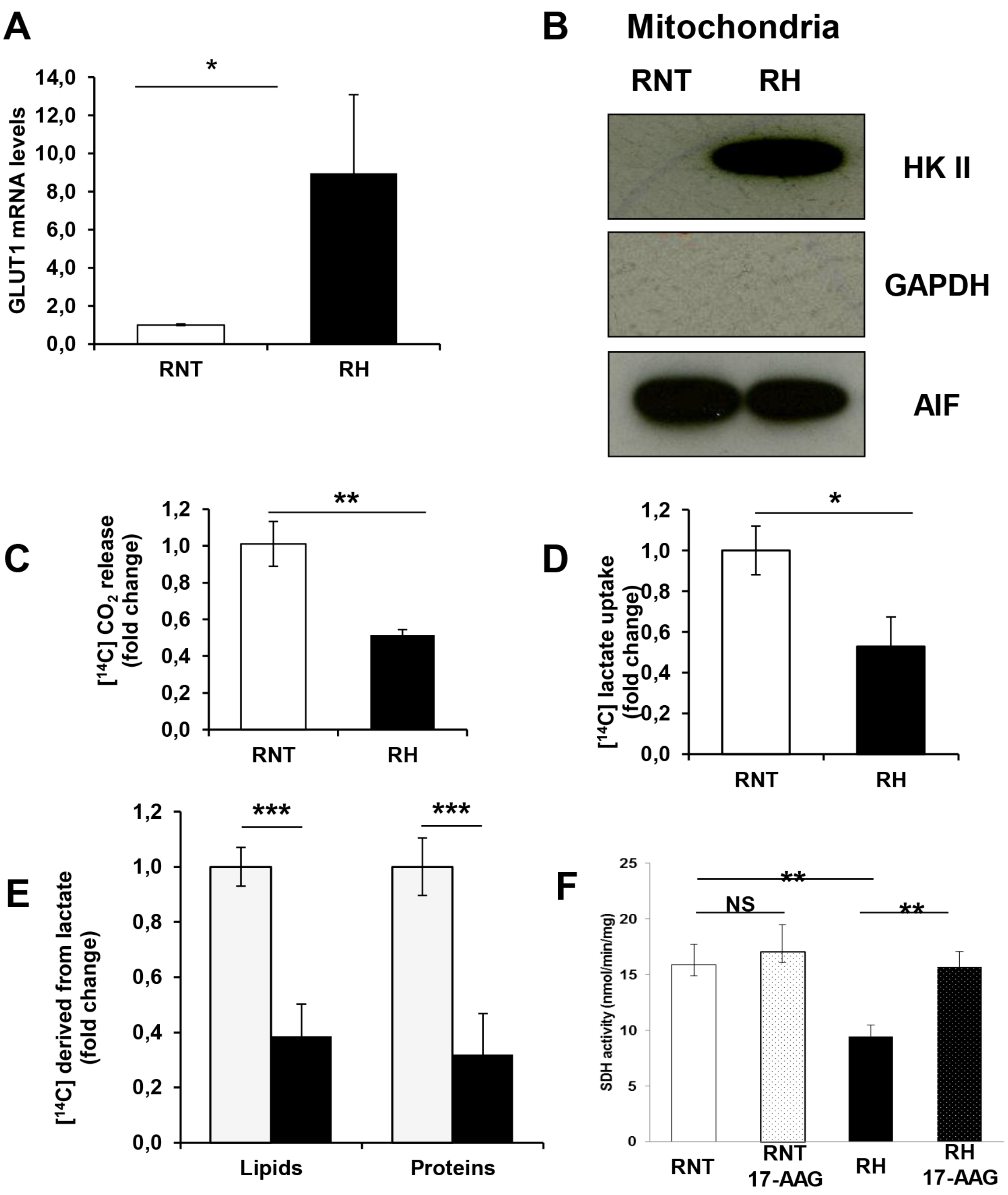


**Supplementary Figure S3: RH cells display a higher glucose metabolism and a lower SDH activity than RNT cells.** **A.** qRT-PCR analysis of GLUT1 mRNA levels in RNT and RH cells. Values are reported as fold-change relative to RNT cells; **P*<0.05. **B.** Western immunoblots analysis of HK II expression on mitochondrial fractions of HCC derived cells (RH) and of the non-tumorigenic hepatocytes (RNT). Blots were probed with anti-AIF and anti-GAPDH antibodies to check for mitochondrial protein load and cytosolic contaminants, respectively. **C.** Radioactive assays using [$^{14}$C]-glucose revealed an impairment of total $CO_2$ production (*i.e.* OXPHOS) in RH cells. **D.** Lactate uptake in RNT and RH cells; **P*<0.05**.** **E.** Radioactive signal derived from lipids and proteins obtained from [$^{14}$C] lactate in RNT and RH cells; ****P*<0.001. **F.** Analysis of the SQR enzymatic activity of SDH in RNT and RH cells; when indicated, cells were treated with the TRAP1 inhibitor 17-AAG (5 µM).

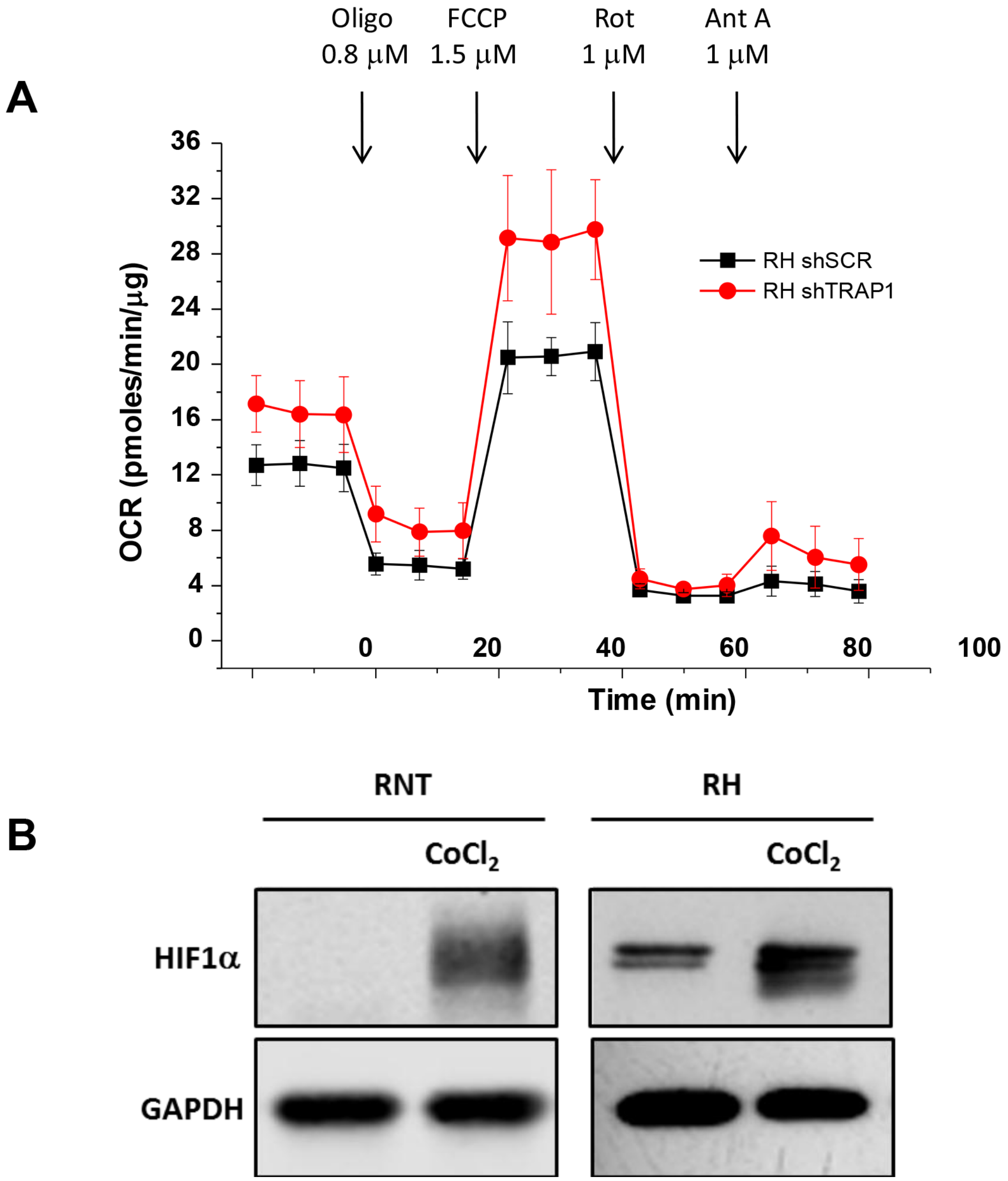


**Supplementary Figure S4: A.** Measurements of oxygen consumption rate (OCR, right) on TRAP1-silenced RH cells. Addition of oligomycin, FCCP, rotenone, and antimycin A was carried out at the indicated times. **B.** WB showing lack of HIF-1α in RNT cells. $CoCl_2$ (200 µM, 6 hours) is used as a positive control for HIF-1α stabilization.

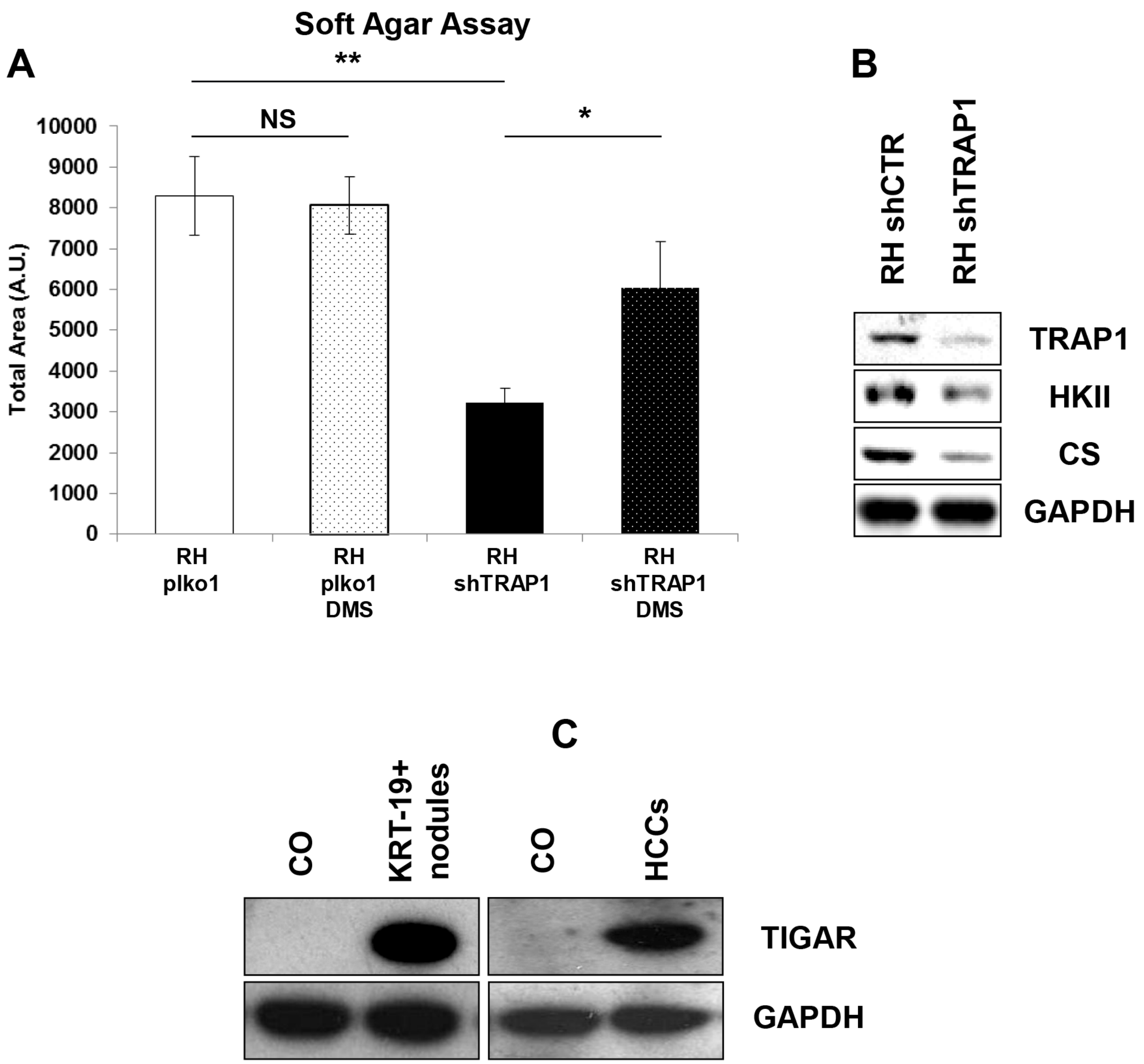


**Supplementary Figure S5: Knocking-down TRAP1 expression inhibits tumorigenicity and the expression of metabolic enzymes in RH cells.** **A.** Soft agar tumorigenesis assays were performed in RH cells following TRAP1 silencing and in combination with di-methyl-succinate (DMS, 5 mM) treatment. Data indicate the total colony area at the $25^{th}$ experimental day. **B.** Western immunoblots showing TRAP1, HK II and CS expression on extracts from RH cells following TRAP1 silencing. GAPDH was used as a loading control. **C.** Western immunoblots showing increased TIGAR expression in KRT-$19^{+}$ nodules and HCC samples compared to control liver (CO). GAPDH was used as a loading control.

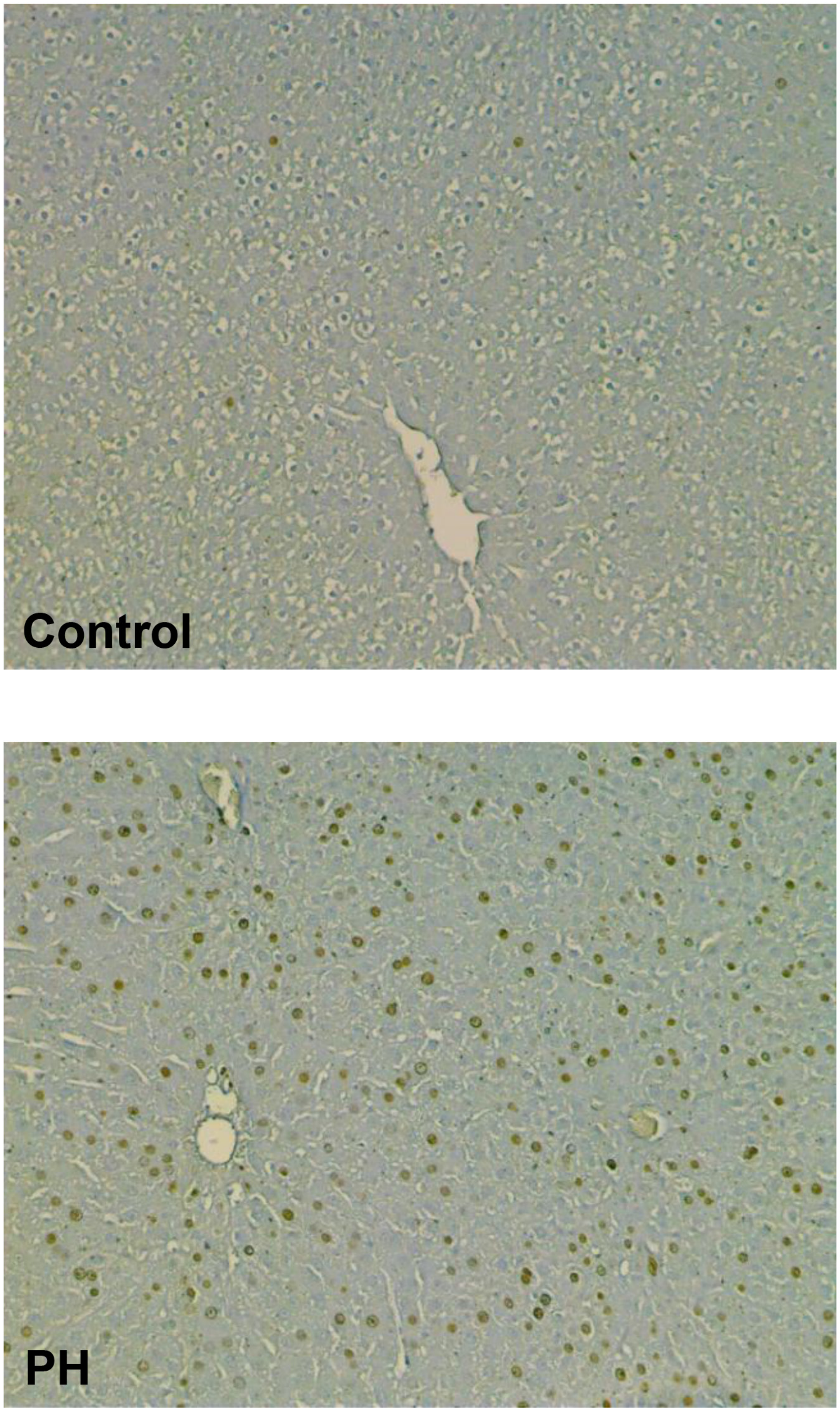


**Supplementary Figure S6: BrdU incorporation in hepatocyte nuclei 24 hours after 2/3 partial hepatectomy (PH).** Microphotograph showing BrdU staining of hepatocyte nuclei (x10). BrdU (100 mg/kg) was given intraperitoneally to animals subjected to surgery and to controls 2 hours prior to sacrifice.

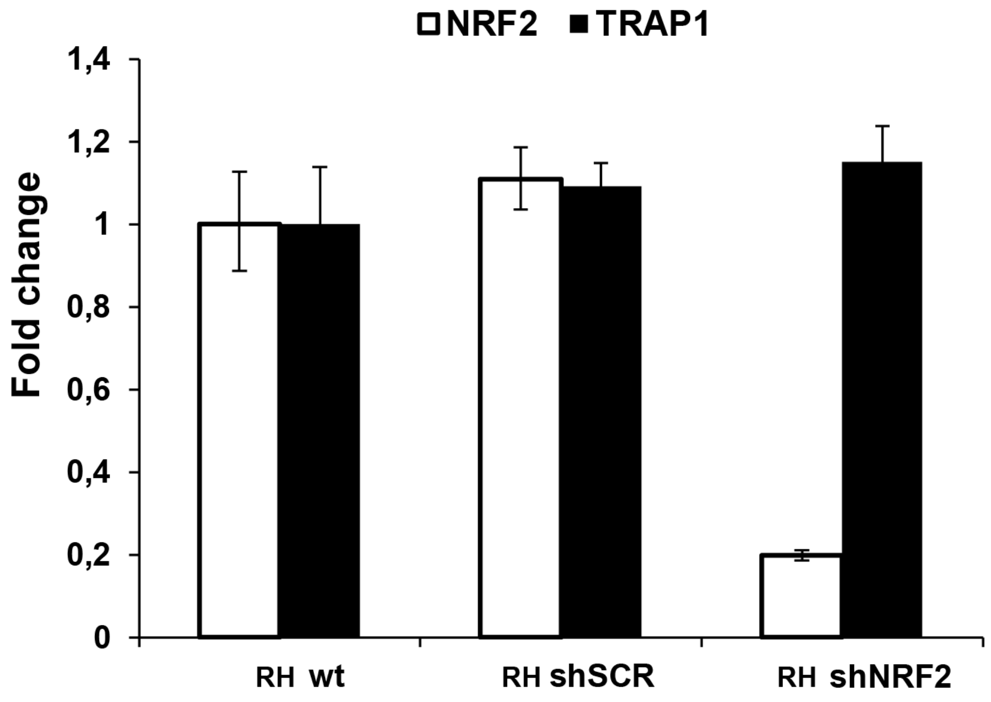


**Supplementary Figure S7: qRT-PCR analysis of NRF2 and TRAP1 mRNA levels in RH cells upon NRF2 silencing.** Values are reported as fold-change relative to cells untransduced wt cells.

**A**

| GENE SYMBOL | FOLD INDUCTION KRT-19+ nodules |
| --- | --- |
| *G6PD* | 5.77 |
| *miR-1* | -5.20 |

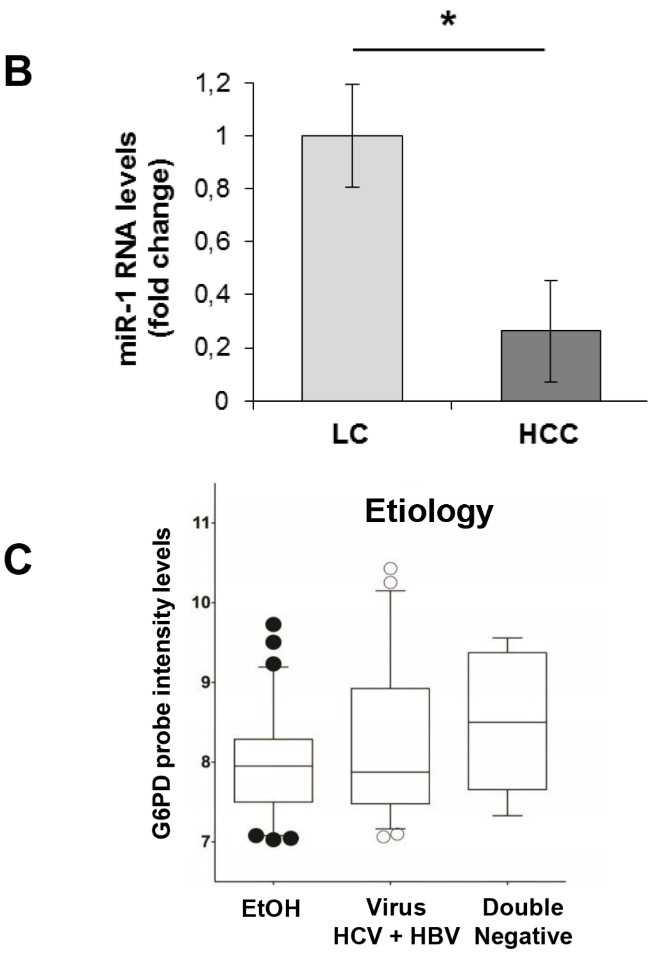


**Supplementary Figure S8: G6PD induction in early preneoplastic lesions and human HCC. A.** Illumina microarray (RatRef-12 V1) and TaqMan Low Density Arrays were used to determine G6PD and miR-1 expression, respectively, in KRT-19$^{+}$ nodules 10 weeks after treatment with DENA. **B.** Changes in miR-1 and G6PD levels in human HCCs. QRT-PCR analysis of miR-1 expression in human resected HCCs (n=59) and their peritumoural cirrhotic liver (Liver cirrhosis, LC; n=59). miR-1 expression is calculated as fold change difference compared to LC. Error bars represent SEM; U6RNA was used as endogenous control; **P*< 0.05. **C.** Stratification of patients according to their etiology did not reveal any significant association with G6PD levels.

**Supplementary Table S1: Study population characteristics.**

**See Supplementary File 1**
