# Supplementary material for: Metabolic reprogramming identifies the most aggressive lesions at early phases of hepatic carcinogenesis
Source: Oncotarget. 2016 Apr 7;7(22):32375–93. doi: 10.18632/oncotarget.8632 (PMC5078020; doi:10.18632/oncotarget.8632)
Supplement: Supplementary file 2 [file oncotarget-07-32375-s002.docx]

SUPPLEMENTAL MATERIAL

SUPPLEMENTARY TABLE 1

A

Patient characteristics, evaluation cohort

**Factors**		**Frequency(%)**
Number of patients	59	
Mean age ± SD	67.8 ± 7.7	
Etiology	HBV	18.6%
	HCV	62.7%
	Alcohol abuse	3.5%
	None	15.2%
Cirrhosis		90%
Serum AFP	> 100 ng/ml	33%
Edmondson grade	I	1.7%
	II	22%
	III	67.8%
	IV	8.5%
Barcelona Clinic Liver Cancer stage	0	1.7%
	A	76.3%
	B	22%
	C	0%
	D	0%

B

Patient characteristics, validation cohort

**Factors**		**Frequency(%)**
Number of patients	59	
Mean age ± SD	64±12	
Etiology	HBV	19%
	HCV	22%
	Alcohol abuse	51%
	None	8%
Cirrhosis		90%
Serum AFP	> 100 ng/ml	33%
Edmondson grade	I	2%
	II	70%
	III	25%
	IV	3%
Barcelona Clinic Liver Cancer stage	0	4%
	A	28%
	B	40%
	C	21%
	D	7%
